# Atlas of mRNA translation and decay for bacteria

**DOI:** 10.1038/s41564-023-01393-z

**Published:** 2023-05-22

**Authors:** Susanne Huch, Lilit Nersisyan, Maria Ropat, Donal Barrett, Mengjun Wu, Jing Wang, Valerie D. Valeriano, Nelli Vardazaryan, Jaime Huerta-Cepas, Wu Wei, Juan Du, Lars M. Steinmetz, Lars Engstrand, Vicent Pelechano

**Affiliations:** 1 SciLifeLab, Department of Microbiology, Tumor and Cell Biology, Karolinska Institutet, Solna, Sweden; 2 Armenian Bioinformatics Institute, Yerevan, Armenia; 3 Institute of Molecular Biology, National Academy of Sciences of Armenia, Yerevan, Armenia; 4 Department of Microbiology, Tumor and Cell Biology, Centre for Translational Microbiome Research, Karolinska Institutet, Stockholm, Sweden; 5 Centro de Biotecnologia y Genomica de Plantas, Universidad Politécnica de Madrid – Instituto Nacional de Investigación y Tecnología Agraria y Alimentaria, Campus de Montegancedo-UPM, Madrid, Spain; 6 Bio-Med Big Data Center, CAS Key Laboratory of Computational Biology, Shanghai Institute of Nutrition and Health, University of Chinese Academy of Sciences, Chinese Academy of Sciences, Shanghai, China; 7 Stanford Genome Technology Center, Stanford University, Palo Alto, CA USA; 8 Department of Genetics, School of Medicine, Stanford University, Stanford, CA USA; 9 European Molecular Biology Laboratory, Genome Biology Unit, Heidelberg, Germany

**Keywords:** Ribosome, Genome-wide analysis of gene expression, Metagenomics, RNA decay

## Abstract

Regulation of messenger RNA stability is pivotal for programmed gene expression in bacteria and is achieved by a myriad of molecular mechanisms. By bulk sequencing of 5′ monophosphorylated mRNA decay intermediates (5′P), we show that cotranslational mRNA degradation is conserved among both Gram-positive and -negative bacteria. We demonstrate that, in species with 5′–3′ exonucleases, the exoribonuclease RNase J tracks the trailing ribosome to produce an in vivo single-nucleotide toeprint of the 5' position of the ribosome. In other species lacking 5′–3′ exonucleases, ribosome positioning alters endonucleolytic cleavage sites. Using our metadegradome (5′P degradome) sequencing approach, we characterize 5′P mRNA decay intermediates in 96 species including *Bacillus subtilis*, *Escherichia coli*, *Synechocystis* spp. and *Prevotella copri* and identify codon- and gene-level ribosome stalling responses to stress and drug treatment. We also apply 5′P sequencing to complex clinical and environmental microbiomes and demonstrate that metadegradome sequencing provides fast, species-specific posttranscriptional characterization of responses to drug or environmental perturbations. Finally we produce a degradome atlas for 96 species to enable analysis of mechanisms of RNA degradation in bacteria. Our work paves the way for the application of metadegradome sequencing to investigation of posttranscriptional regulation in unculturable species and complex microbial communities.

## Abstract (web summary)

Metadegradome sequencing maps 5′P mRNA decay intermediates in complex samples and 96 isolated bacterial species, to identify codon- and gene-level ribosome stalling responses to stress and drug treatment.

## Main

Messenger RNA (mRNA) degradation and translation are closely connected, and changes in ribosome dynamics directly modulate messenger RNA stability^1–4^. In eukaryotes, protein synthesis and mRNA decay are connected by the 5′–3′ exonuclease Xrn1, which follows the last translating ribosome producing an in vivo toeprint of its position^5,6^. RNA degradation in bacteria enables adaptation to changing environments, but our understanding of RNA degradation in bacteria has been hampered by the diversity of RNA degradation mechanisms. Bacterial RNA degradation was thought to initiate via endonucleolytic cleavage followed by 3′–5′ degradation^7–10^. Although 5′–3′ RNA exonucleolytic activity of RNase J in *Bacillus subtilis*^11^ and its homologues in other species^9^ has been reported, and translational efficiency is known to modulate mRNA stability in bacteria^3,12^, we do not understand how cotranslational mRNA degradation shapes the bacterial degradome or whether this process differs in species with divergent mRNA degradation machinery.

To better understand the regulation of RNA biology in bacteria, we investigated mRNA degradation in reference bacterial strains and complex microbiomes using optimized 5' monophosphorylated (5′P) mRNA decay intermediates (degradome) sequencing (5PSeq). We explored the degree to which naturally occurring 5′P reflects in vivo ribosome dynamics and characterize the 5′P degradome in response to environmental stress and drug treatment in reference strains and complex faecal cultures. We analysed mRNA degradation patterns in 96 culturable and unculturable species, including the model organism *B. subtilis*, and report our findings here.

## Cotranslational mRNA degradation in bacteria

**Fig. 1:**
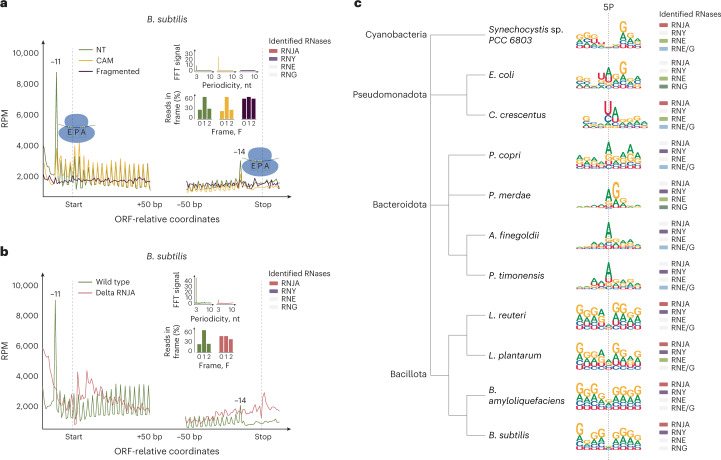
5′P mRNA sequencing as a proxy for in vivo prokaryotic ribosome dynamics. **a**, Metacounts (5PSeq reads per million (RPM)) of 5' mapping positions relative to translation initiation and termination sites of ORFs from *B. subtilis*. No treatment (NT) control (green), CAM-treated (yellow) and randomly fragmented (in red) are shown. Identified RNases are highlighted in the upper right corner (those unidentified are coloured light grey). Original 5PSeq positions are reported (no P-site correction was applied). FFT for the observed periodicity and a histogram depicting relative 5PSeq frame protection for all codons are also shown. **b**, 5′P metacount for ∆*rnjA* strain, as in **a**. **c**, Cleavage motif preference at 5′P sites and ± four bases for representative species from the phyla Bacillota, Cyanobacteria, Pseudomonadota and Bacteroidota, computed with the Shannon entropy-based measure of nucleotide contribution as implemented in the R package ggseqlogo^64^. Right, the presence of RNases identified in each species is highlighted. RNE/G (ribonuclease E/G family proteins) indicates the presence of the conserved catalytic domain between both ribonucleases. EPA, tRNA binding sites E, P, and A on the ribosome.

**Extended Data Fig. 1:**
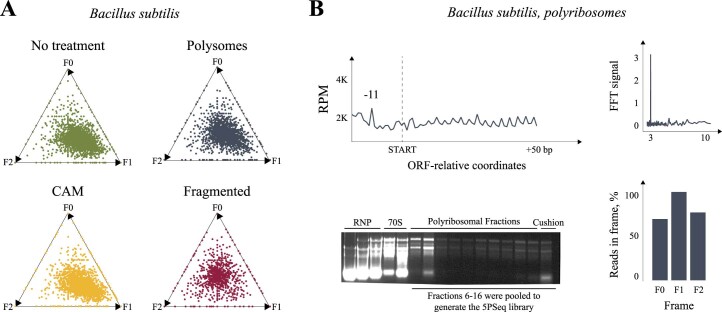
5’P 3-nt periodicity is associated with co-translation mRNA decay. **A**, Gene-specific 3-nt protection for *B. subtilis* as reported by fivepseq^14^. Each point corresponds to a gene and the proximity to the triangle boundaries (F0, F1, or F2) their preferential protection frame. NT control cells are shown in green, polyribosome-associated mRNA degradation intermediates in blue, CAM treated cells in yellow, and randomly fragmented in red. **B**, 5PSeq metagene analysis of *B. subtilis* after polyribosome fraction isolation (as in Fig. 1).

We analysed the 5′P mRNA degradome in individual species and complex bacterial communities using optimized 5PSeq)^5,13–15^ (Supplementary Tables 1 and 2 and Methods). First we investigated the 5′P degradome of open reading frames (ORFs) in species with 5′–3′ decay. *B. subtilis* encodes the 5′–3′ exonuclease RNase J^11^. Although RNase J is not homologous to the eukaryotic XRN1 we hypothesized that, via its 5′–3′ exonuclease activity, it could ‘chase’ the last translating ribosome in mRNAs undergoing cotranslational decay, similar to XRN1 in yeast^5,16^. Our analysis revealed a 3 nucleotide (nt) periodicity of 5′P counts (Fig. 1a) with a 5′P preference for the second nucleotide of each codon (F1), at both the metagene and single-gene levels (Fig. 1a and Extended Data Fig. 1a). 5′P degradation intermediates accumulate 11 and 14 nt upstream of translation start and stop sites, respectively, as expected from slow initiating and terminating ribosomes. This pattern is analogous to sites –14 nt from the start and –17 nt from the stop in budding yeast^5,15^. This 3 nt smaller protection size can be explained by the known difference in ribosome sizes between eukaryotes and bacteria^17^. We confirmed the association of 5′P decay intermediates with translating ribosomes by the application of 5PSeq to polyribosomal fractions of sucrose density gradients, and observed similar patterns (Extended Data Fig. 1b). To demonstrate its biological origin, we confirmed that in vitro fragmentation of the same RNA eliminated the observed in vivo 3 nt periodicity by >99.5% (Fast Fourier transform (FFT) signal dropping to 0.11) and by start/stop-associated toeprints (Fig. 1a). Finally, to investigate whether there is a role for RNase J in this process, we repeated the analysis in a *B. subtilis* RNase JA deletion (*rnjA*) strain. This revealed that RNase JA activity underpins the observed 5′P distribution pattern resulting from cotranslational mRNA degradation (Fig. 1b).

**Extended Data Fig. 2:**
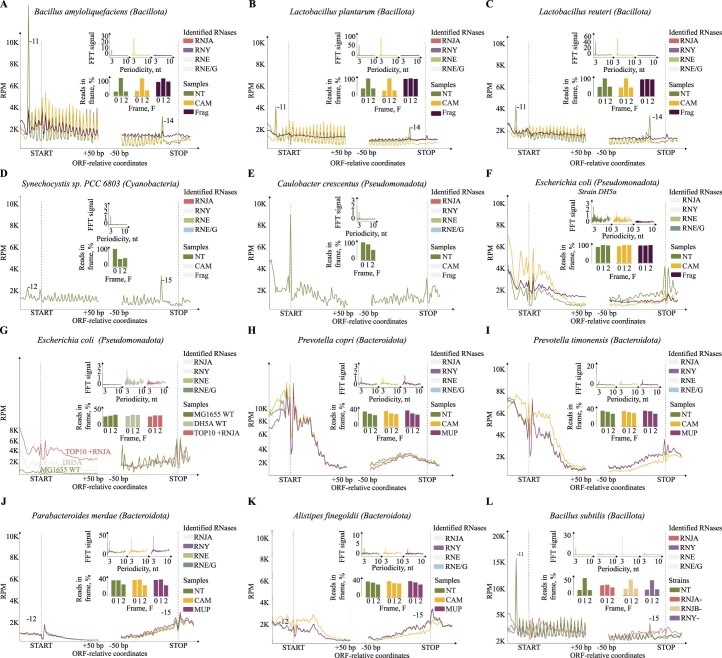
Ribosome-associated 3-nt periodicity can be found in multiple bacterial species. **A-L**, Metagene analysis for multiple species displaying metagene 5PSeq protection, Fast Fourier Transform (FFT), relative frame protection, and identified RNases (as in Fig. 1).

Next, we investigated species with or without 5'–3' RNA exonucleases annotated in their genome (Extended Data Fig. 2)^9^ to assess the role of other nucleases in mRNA degradation. First, we tested Bacillota (*Bacillus amyloloquefaciens, Lactobacillus plantarum, Lactobacillus reuteri*), Cyanobacteria (*Synechocystis* sp. PCC 6803) and Pseudomonadota (*Caulobacter crescentus*), all of which encode RNase J. We observed a clear 3 nt periodicity pattern along full-length coding regions, with 5′P reads frequently accumulating at the second nucleotide (F1) of each codon and 11 and 14 nt upstream of the start and stop codons, respectively (Extended Data Fig. 2), as observed for *B. subtilis* (Fig. 1a). Interestingly, in *Synechocystis* sp. PCC 6803 the ribosome protection pattern was displaced by one extra nucleotide (that is, accumulation at –12 nt from the start and –15 nt from the stop codons) (Extended Data Fig. 2d). This might be explained by either a different ribosome protection size or conformation, or the presence of potential cofactors^18,19^. We also tested Pseudomonadota species, including *Escherichia coli* (DH5α and MG1655), and anaerobic Bacteroidota (*Alistipes finegoldii*, *Prevotella copri*, *Prevotella timonensis* and *Parabacteroides merdae*), none of which have 5'–3' exonuclease activity. We observed a 3 nt periodicity pattern in the studied Bacteroidota, which have RNE/G protein family (RNE/G) homologues^20^ and RNase Y. In *E. coli*, which has RNase E rather than RNase Y, we observed a subtle 3 nt periodicity (Extended Data Fig. 2f) that was less noticeable in strain MG1655, where fewer 5′P reads accumulate in the coding region (Extended Data Fig. 2g). In contrast to species with 5'–3' exonuclease, those lacking RNase J accumulate 5′P endonucleolytic sites around the start and stop codons. Additionally we observed 5′P cleavage overlapping the start codon itself in most species, and different from the expected ribosome protection at 11 nt upstream. Cleavage associated with the start codon was particularly clear in *C. crescentus*. We propose that there is a relationship between translation initiation and mRNA decay, where endonucleolytic cleavages often occur at the start codons (Extended Data Fig. 2).

**Extended Data Fig. 3:**
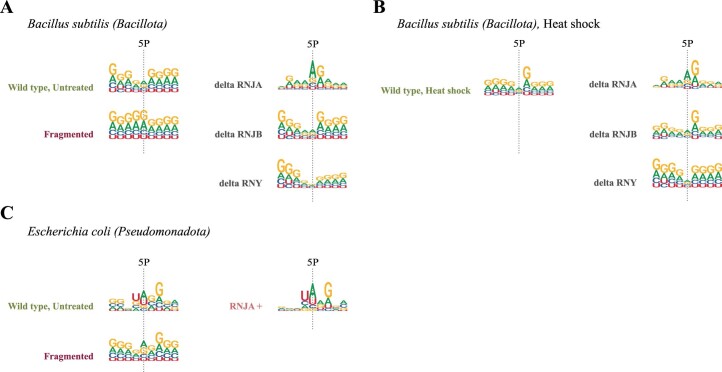
Cleavage motif preference at 5´P sites + /- 4 bases. **A**, NT and fragmented controls of *B. subtilis* wild type strains (168 trpC2), as well as rnjA, rnjB knock-out strains. **B** Wild type and RNase mutant *B. subtilis* strains under heat shock. **C**. NT control and fragmented samples of *E. coli Dh5α* strain and the TOP10 with heterologous expression of *S. pyogenes* RNJA. Cleavage motif preference was computed with Shannon entropy-based measure of nucleotide contribution (as in Fig. 1C).

The observed 3 nt periodicity might have originated from in vivo 5'–3' single-nucleotide toeprinting of the ribosome position, as reported in eukaryotes^5^. Alternatively, it could be a consequence of the interaction among endonuclease-specific cleavage preferences, differential mRNA accessibility and non-random distribution of nucleotides along the coding regions. Previous work has investigated the sequence preference of RNase E^21,22^ and RNase Y^23,24^ in selected species, so we opted to include a diverse set of species with different RNA degradation machinery to expand the scope of species covered (Fig. 1c). The presence of RNase J and E without Y in *Synechocystis* sp.^25^ and *C. crescentus* is associated with 5′P mRNA degradation intermediates with a preference for an A preceded by a U (–1 U/+1 A). The presence of RNase Y and RNE/G protein homologues in Bacteroidota suggests a preference for +1 A. In Bacillota, where 5'–3' exonucleolytic activity dominates, 5′P mRNA degradation intermediates present more homogenous sequence motifs. It is feasible that RNase J trims endonucleolytically generated cleavage sites. Consistent with this hypothesis, a 5′P preference for A emerges in the *B. subtilis rnjA* deletion mutant (Extended Data Fig. 3a), similar to Bacteroidota where the endonuclease RNase Y is the main driver of RNA decay (Fig. 1c)^26,27^. We tested the effect of RNase JB (*rnjB*) and RNase Y (*rny*) deletions in *B. subtilis* and found that their contribution to the observed pattern was minimal (Extended Data Fig. 3a). Interestingly, when performing the same analysis after heat shock (HS) we observed a clear increase in +1 G preference in all samples (Extended Data Fig. 3b). This suggests that the interplay between RNases and ribosomal position is dynamic and that it can be altered in response to environmental challenges. We also tested whether the heterologous expression of *Streptococcus pyogenes* *rnjA* was sufficient to alter the endonucleolytic pattern observed in *E. coli*. However, even when *rnjA* was expressed at mRNA level as measured by 5PSeq, the pattern (–1 U/+1 A/+3 G) was maintained (Extended Data Fig. 3c), suggesting that RNase E activity still dominates mRNA decay. To confirm that the observed patterns are caused by biological mechanisms, we demonstrate a loss of 5′P preference following in vitro RNA fragmentation in samples from *B. subtilis* and *E. coli* (Extended Data Fig. 3a,c). We conclude that the sequence preference of the naturally present 5′P mRNA degradation intermediates is conserved across phylogenetically related species and is shaped by the crosstalk between RNA degradation pathways.

## 5′P mRNA degradation as proxy for ribosome position

**Fig. 2:**
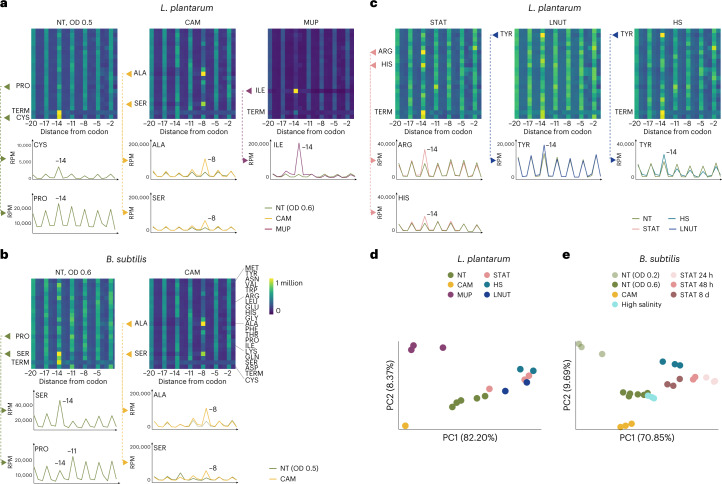
Codon-specific ribosome-pausing patterns under stress and antibiotic treatment. **a**–**c**, Heatmaps for amino acid-specific 5′P RNA coverage colour-coded blue (low) to yellow (high). The distance from specific amino acids is indicated as the number of nucleotides. Ribosomes paused at –14 indicate an A-site stall. Selected amino acids are shown also as line plots. **a**, *L. plantarum* pauses following CAM (yellow) and MUP (purple) treatment compared with the NT control sample collected at OD_600_ = 0.6–0.8 (OD = 0.6). **b**, *B. subtilis* context-specific CAM pauses induced by penultimate amino acid (of peptide chain) shown at the –8 position (CAM, yellow). **c**, *L. plantarum* pauses following stress treatment. **d**,**e**, PCA of ribosome protection phenotype (Methods) distinguishes between stress and drug treatment in *L. plantarum* (**d**) and *B. subtilis* (**e**). The separate clusters of untreated *B. subtilis* can be explained by differences in their growth phase at harvest (OD_600_ 0.2–0.3 and 0.6–0.8, respectively).

**Extended Data Fig. 4:**
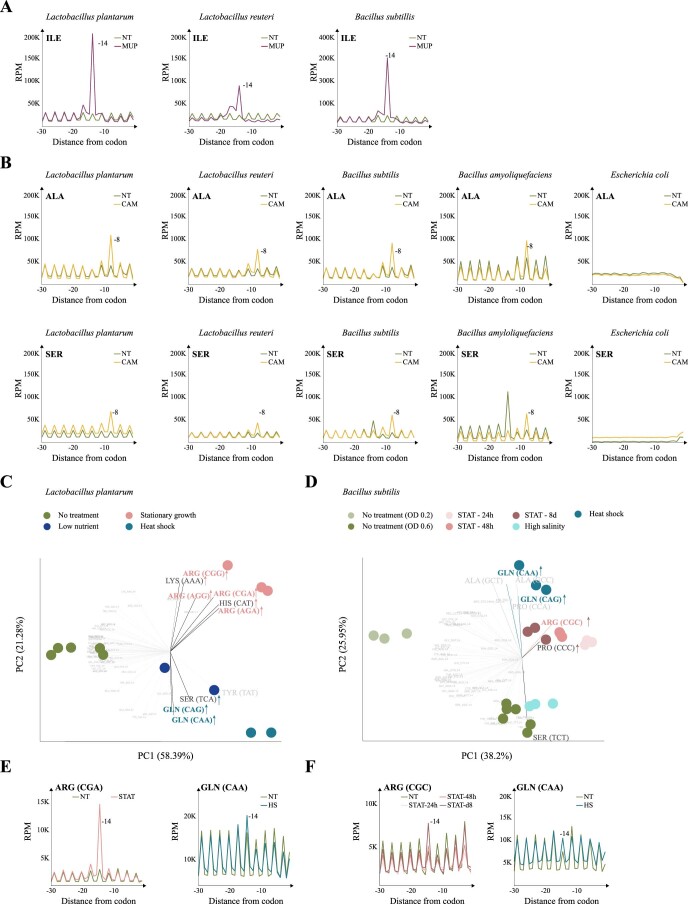
Species- and codon-specific ribosome pauses in response to stress or antibiotic treatment. Line plots showing amino acid-specific ribosome pauses as measured by fivepseq. **A**, Isoleucine (Ile) pause (-14 nt) comparing NT and MUP treatment in *L. plantarum*, *L. reuteri* and *B. subtilis*. **B**, The relative context-specific Alanine (Ala) and Serine (Ser) ribosome pauses (-8 nt) as measured by fivepseq for multiple species in response to CAM. Only in *E. coli*, which lacks RNase J, the 5´P degradome profile does not provide single-nucleotide resolution information of the ribosome stall. **C-D**, Principal component analysis plots based on 5P counts 14 nt upstream from each codon for *L. plantarum* and *B. subtilis* in stress conditions. The NT control samples of *B. subtilis* have been collected at different growths phases (OD_600_ 0.2-0.3 and 0.6-0.8 respectively). The contribution of each codon is shown with gray loading vectors. The features with the longest loadings corresponding to those with highest absolute weight in the PC1 and PC2 are highlighted. The up arrows in labels indicate that the 5P counts increase in stress conditions for the codon. Codons of common amino acids between *L. plantarum* and *B. subtilis* are highlighted with stress-specific colours. **E-F**, relative Arg and Gln pauses (-14 nt) in stationary phase growth for *L. plantarum* (27 hours) and *B. subtilis* (8 days).

To investigate how ribosomes affect 5′P distribution we perturbed ribosome dynamics using chloramphenicol (CAM). When ribosomes stalled at elongation we observed an increased 3 nt ribosome protection pattern in *B. subtilis*, *L. plantarum*, *L. reuteri* and *B. amyloliquefaciens* (Fig. 1a and Extended Data Fig. 2a,c). We also observed a relative excess of 5′P degradation intermediates around the 5′ regions of ORFs and a decrease in protection around the 3′ regions, consistent with specific inhibition of translation elongation rather than initiation or termination^17^. 5′P accumulation in the 5′ regions of ORFs was also evident in species without 5'–3' exonuclease activity, in which endonucleolytic activity cannot be expected to provide single-nucleotide-resolution information regarding ribosome position (Extended Data Fig. 2f–k). Our results suggest that ribosome stalling after CAM treatment may limit the in vivo accessibility of endonucleases and affect the 5′P mRNA degradome. Next, we considered whether the abundance of 5′P mRNA decay intermediates could serve as a proxy for codon-specific ribosome pauses in species containing RNase J. By alignment of 5′P reads to each respective amino acid we generated amino acid-specific metagene profiles (Fig. 2 and Extended Data Fig. 4). In *L. plantarum* this showed a clear 5′P accumulation associated with slow ribosomes at the stop and Cys codons, probably associated with limited availability of free Cys in the growth medium (Fig. 2a)^28,29^. Next, we perturbed the translation process and tested the effects on 5′P accumulation. First, we tested our ability to detect codon-specific pauses after 10 min treatment with mupirocin (MUP), an antibiotic targeting Ile tRNA synthetase. MUP treatment led to ribosome stalls at Ile codons and a clear accumulation of 5′P reads 14 nt upstream of those in *L. plantarum*, *L. reuteri* and *B. subtilis* (A-site stall; Fig. 2a and Extended Data Fig. 4a). This suggests that ribosome positions shape the bacterial degradome. Next, we investigated codon-specific pauses associated with 5 min CAM treatment in *B. subtillis*, *L. plantarum*, *L. reuteri* and *B. amyloliquefaciens* (Fig. 2a,b and Extended Data Fig. 4b). In addition to a general inhibition of translation elongation, CAM also leads to context-specific accumulation of ribosomes^30,31^. Namely, CAM treatment of *E. coli* has previously been reported to increase ribosome stalling when Ala (and, less frequently, Ser, Thr or Gly) is positioned in the E site. Using 5PSeq, we also identified CAM-induced ribosome stalling 8 nt upstream of Ala and Ser in the tested species with RNase J (Fig. 2a,b and Extended Data Fig. 4b). We conclude that context-specific ribosome stalls following CAM treatment are conserved in bacteria. Additionally, our results show that 5′P signatures can be used as molecular reporters for antibiotics that target the translation process in species with RNase J.

Next, we studied 5′P RNA degradation profiles under stress conditions in *B. subtillis*, *L. plantarum* and *L. reuteri* (Fig. 2c and Extended Data Fig. 4). We identified clear ribosome pauses in *L. plantarum* at Tyr codons under both heat shock and low nutrient (LNUT) conditions, and at His and Arg codons in stationary growth (STAT; Fig. 2c). 5P-seq provides an overview of the ribosome-dependent accumulation of 5′P degradation intermediates at all amino acid positions, and we used these data to generate a general amino acid-specific ‘ribosome protection phenotype’ for each sample (Methods). Combining this phenotype with principal component analysis (PCA) enabled us to separate control, stressed and drug-treated samples in *L. plantarum* (Fig. 2d). Similarly, the same strategy separates drug treatment, stress conditions and time-course measurement of the stationary phase in *B. subtilis* (24 h, 48 h and 8 d) (Fig. 2e). Thus, codon-specific ribosome protection phenotypes can be leveraged to distinguish drug treatment and other stress conditions. To analyse codon-specific ribosome stalls in response to environmental changes, we focused on 5′P reads associated with ribosome A-site stalls (Extended Data Fig. 4c,d). After heat shock in *L. plantarum* we found that Gln codons have higher 5′P coverage, suggesting codon-specific ribosome stalls. In stationary growth, Arg codons (CGG, CGA, AGG and AGA, but less so than CGG and CGU) were associated with ribosome stalls (Extended Data Fig. 4e). Interestingly, when applying the same strategy to *B. subtilis*. (Extended Data Fig. 4d) we observed that heat shock was also associated with ribosome stalls at Gln codons (CAA and CAG) and stationary growth with stalls at Arg codons (CGC) (Extended Data Fig. 4f). This suggests that environmental challenges can result in similar codon-specific ribosome stalls, which may result from either stress-specific defects of tRNA processing and biosynthesis^32,33^ or changes in amino acid pools^29,34,35^.

**Extended Data Fig. 5:**
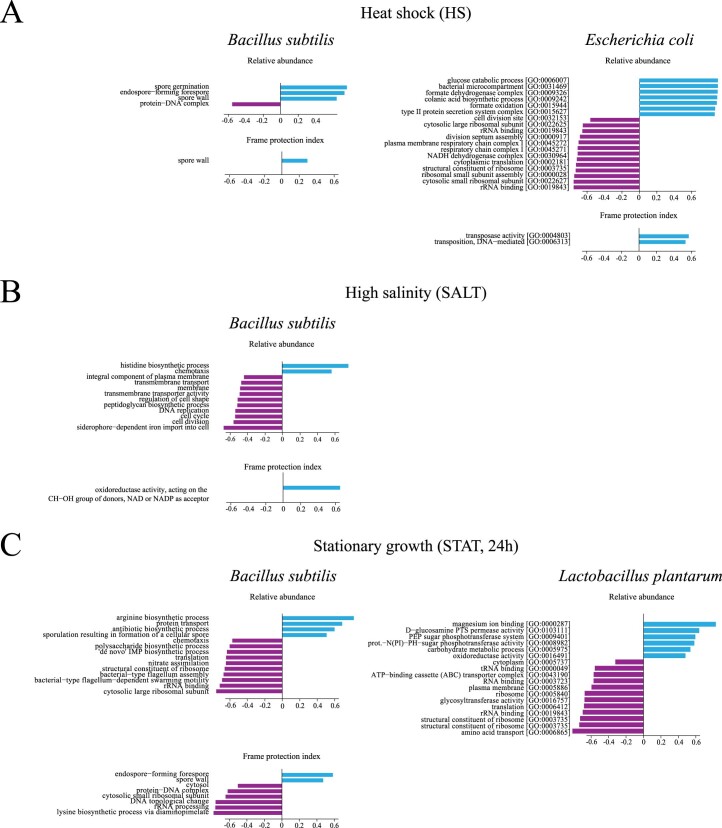
Gene set enrichment analysis of Gene Ontology functional sets in stress conditions. Either the log2 fold change of the reads undergoing degradation in stress compared to untreated samples (relative abundance) or the log2 fold change of the frame protection index (FPI) is considered when performing the enrichment analysis. Only results with false discovery rate of <0.05 are shown. **A**. *B. subtilis* and *E. coli* in heat shock. **B**. *B. subtilis* in high salinity. **C**. *B. subtilis and L. plantarum* in stationary growth.

In addition to global patterns, 5PSeq also provides information regarding gene-specific ribosome stalls. We measured the relative strength of 3 nt protection patterns across genes (gene-specific frame protection index (FPI); Methods). This provides a measure of the relative stalling of the last translating ribosome independent of mRNA abundance for each gene (that is, comparing peaks and valleys along the mRNA sequence). Drug and stress treatments induce changes in the FPI of individual genes involved in bacterial responses (Extended Data Fig. 5 and Supplementary Table 3). For example, heat shock leads to increased FPI (higher 3 nt periodicity) for mRNAs associated with the spore wall (false discovery rate (FDR) < 0.022), and also salt stress, with oxidoreductase activity (FDR < 0.011). During stationary growth, FPI of genes related to the spore wall increased (FDR < 2.2 × 10^–16^) while that of genes involved in Lys biosynthesis and ribosomal RNA processing decreased (FDR < 0.024 and < 0.044, respectively). Interestingly, when exposed to CAM or MUP, the relative FPI of ribosomal protein genes clearly decreased (FDR < 2.2 × 10^–16^), suggesting a relative change in ribosome protection with respect to the remainder of the transcriptome in response to the drugs. These findings demonstrate that 5P-seq can inform about ribosome position and translation regulation during stress also at the gene-specific level, and detect rapid phenotypic translational responses to drug treatment.

## 5′P RNA degradome is controlled by environmental conditions

**Extended Data Fig. 6:**
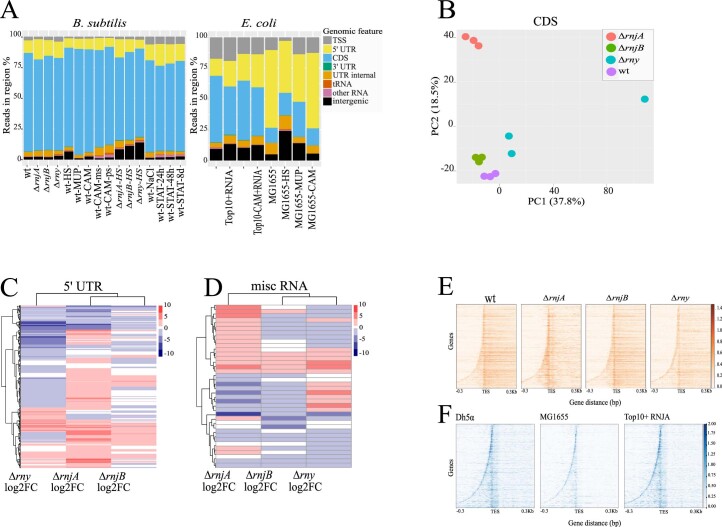
Analysis of degradome fragment in respect to genomic features. **A**, Barplots showing the percentage (%) (Y-axis) of 5’ P reads overlapping a given genic annotation feature for *B. subtilis* (left) and *E. coli* strains (right panel) under normal and perturbed conditions. Contaminant rRNA reads were excluded from the analysis. **B**, Principal component analysis based on library size normalized 5’ P reads on the natural logarithmic scale of *B. subtilis* control and different RNase knockdowns. Axes represent principal components 1 and 2. **C**, Heat map showing the log_2_ fold change in 5’ P reads abundance for miscRNAs for RNase knockouts vs the wild type *B. subtilis* strain. **D**, as C but for 5' UTRs. **E**, Heat maps showing the strand-specific coverage of library size normalized 5’ P reads centreing around the end site (TES) of 5' UTRs (in this case the start codon) for *B. subtilis* control and different RNase knockouts. Each heat map row shows one 5' UTR. Dashed line indicates the start site of 5' UTRs (that is, TSSs). **F**, as E, but for *E.coli*.

Quantification of the relative abundance of mRNA degradation intermediates can be performed using 5PSeq. We investigated how the degradome was altered globally in multiple species using different perturbations. First, we investigated the genome-wide abundance of 5′P RNA molecules (Extended Data Fig. 6a). 5′P RNA distribution across the *B. subtilis* chromosome was relatively stable for all tested RNase mutants, but we observed an increase in RNAs originating from intragenic regions after heat shock. In *E. coli* we observed differences between strains, with 5′P RNA molecules preferentially associated with the coding region in DH5α and with 5′ untranslated regions (UTRs) in MG1655, in agreement with the observed differences in 5′P RNA around coding regions (Extended Data Fig. 2). We also observed increased 5′P RNA from intergenic regions in *E. coli*. Next, we investigated variation in 5′P degradation intermediates at the single-gene level, a measure that integrates the abundance of each mature RNA with the likelihood of decay (Extended Data Fig. 6 and Supplementary Table 3). We quantified the gene-specific abundance of 5′P mRNA fragments in *B. subtilis* and showed that heat shock increased degradation intermediates for spore germination genes (FDR < 7.68 × 10^–4^), salt stress and His biosynthesis (FDR < 2.2 × 10^–16^). Stationary growth led to a decrease in 5′P mRNA fragments associated with translation in both *B. subtilis* and *L. plantarum* (FDR < 2.2 × 10^–16^). In *E. coli*, heat shock led to a decrease in degradation intermediates associated with translation (FDR < 2.2 × 10^–16^) and an increase in those associated with glucose catabolism (FDR < 0.016). Next, we examined how specific RNases in *B. subtilis* affect the abundance of gene-specific 5′P mRNA degradome fragments. Consistent with our metagene analysis, *rnjA* deletion separated from the wild-type strain and *rnjB and rnY* deletion mutants in a PCA of gene-specific abundance of mRNA degradation intermediates (Extended Data Fig. 6b and Supplementary Table 4). We also observed a decrease in degradation intermediates associated with translation (FDR < 2.2 × 10^–16^) (Supplementary Table 3). *rnjB* deletion led to a decrease in degradation intermediates associated with the phosphoenolpyruvate-dependent sugar phosphotransferase system (FDR < 2.2 × 10^–16^) and *rny* deletion led to a decrease in degradation intermediates associated with chemotaxis (FDR < 2.2 × 10^–16^).

To characterize the regulatory functions of RNases we investigated the effect of their depletion on the abundance of regulatory RNAs and 5' UTRs (Extended Data Fig. 6c,d and Supplementary Table 4). *rnjA* deletion led to an increase in histidyl-tRNA synthetase 5′ UTR (FDR < 8.86 × 10^–7^), the T-box riboswitch specific of Ser tRNA ligase (FDR < 3.18 × 10^–7^) and the small regulatory RNA *roxS* (FDR < 7.9 × 10^–4^) among many others (see Extended Data Fig. 6c,d and Supplementary Table 4 for details and other RNases). Interestingly, in addition to coverage in the coding region, we also identified 5′P peaks overlapping the transcription start sites in *B. subtilis* and *E. coli* (Extended Data Fig. 6e,f).

## 5PSeq characterizes ribosome positions in microbiome samples

**Extended Data Fig. 7:**
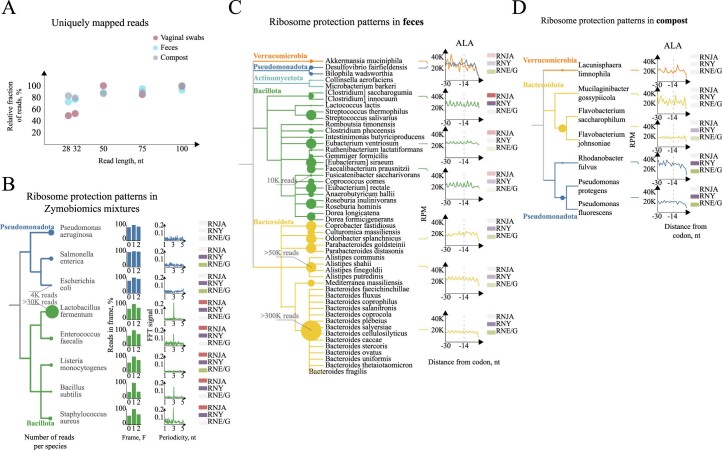
Longer ribosome protected 5PSeq reads enable better species-specific assignment in complex microbiomes. **A**, Relative number of uniquely mapped reads (scaled within each sample) as a function of used read length (trimmed computationally). **B**, 5PSeq analysis from frozen cell suspension from the ZymoBIOMICS Microbial Community Standard (intended for DNA analysis). The numbers of assigned reads to each species are marked in circles. Relative frame protection and Fast Fourier Transform (FFT), and presence/absence of enzymes as in Fig. 1. **C**, 5PSeq analysis from fecal microbiomes. The numbers of assigned reads are marked in circles. Example of *in vivo* amino acid-specific (Ala) 3-nt ribosome protection periodicity for selected species. **D**, same for compost microbiome.

Having shown that 5PSeq maps ribosome dynamics in bacteria, we next applied it to mixed samples. Although human microbiomes have a role in health and disease^36,37^, our understanding of microbiome posttranscriptional regulation is limited. Ribosome profiling, based on polyribosome fractionation followed by in vitro ribosome footprinting and sequencing, has been used to map ribosome position^19^ but its application to the microbiome is technically challenging^38^ and limited by the small size of ribosome protection fragments after in vitro RNA digestion (~23–24 nt)^17,39^. Degradome sequencing has potential advantages, including (1) longer, in vivo generated ribosome-protected fragments, which may enable the resolution of closely related species in a sample (Extended Data Fig. 7a); (2) simplicity, without the need for subcellular fractionation^40^; (3) being applicable to stored RNA samples^5,41^; and (4) providing information about in vivo ribosome position, codon-specific ribosome stalls and amino acid limitations in species containing RNase J, which is estimated to represent ∼60% of bacterial species^9^.

**Fig. 3:**
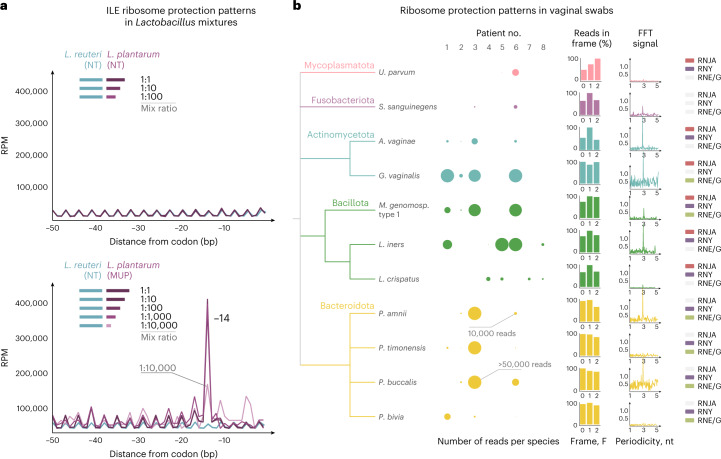
Species- and codon-specific ribosome pauses in complex samples. **a**, Line plots showing 5P|Seq metagene abundance with respect to Ile codons for *L. reuteri* (light blue) and *L. plantarum* (purple) mixed at different ratios (1:1–1:10,000; *L. plantarum* versus *L.reuteri*). MUP treatment of *L. plantarum* (bottom) led to clear Ile pauses with respect to the *L. reuteri* NT control. *L. plantarum* and *L. reuteri* have 63–80% of genome similarity between their coding regions. **b**, 5PSeq analysis of previously isolated vaginal microbiomes. The numbers of reads assigned to each species and healthy donors are denoted by circles. Relative frame protection and FFT, as well as the presence/absence of selected RNases, as in Fig. 1.

To demonstrate the applicability of 5PSeq to bacterial communities, we first analysed 5′P mRNA degradation intermediates in a mix of two closely related species, *L. reuteri* and *L. plantarum*. We pooled different amounts of RNA from MUP-treated *L. plantarum* with untreated *L. reuteri* (1:1–1:10,000; Fig. 3a). MUP-associated Ile ribosome pauses can be detected even at the 1:10,000 ratio (0.01% abundance). The limit is driven by sequencing depth (that is, in the 1:10,000 mix only 82 reads were mapped to *L. plantarum* coding sequences). Having confirmed our ability to detect species-specific perturbations, we next investigated ribosome dynamics in defined microbial community standards (Extended Data Fig. 7b). We then investigated clinical and environmental microbiome samples, studying the metadegradome of vaginal microbiomes using previously isolated RNA samples^42^. In addition to RNA-based indication of species abundance, we obtained degradation profiles for most identified species (Fig. 3b). These data enabled the investigation of in vivo ribosome protection phenotypes across species and patients. Finally we applied our method to more complex microbiomes (human faecal microbiome and compost; Extended Data Fig. 7c,d).

## Detection of species-specific perturbations in complex microbiomes

**Fig. 4:**
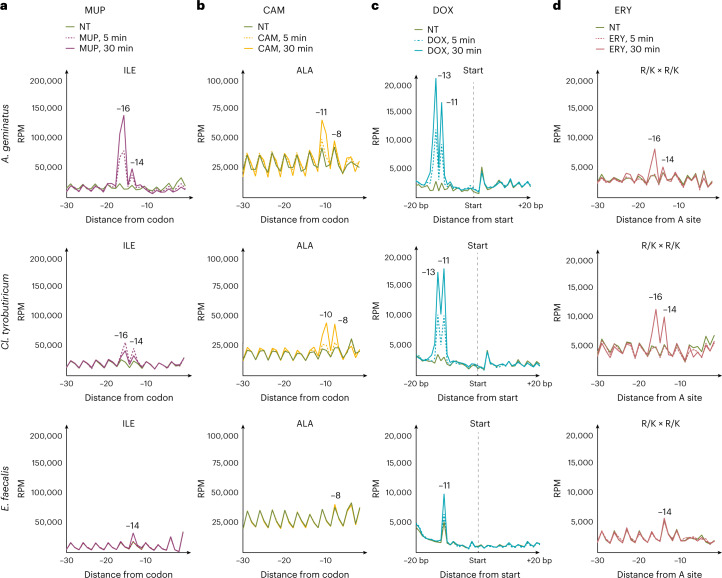
Translational responses to drug perturbations in faecal microbiomes. **a**–**d**, Line plots showing 5PSeq metagene abundance with respect to selected features. Distance from specific amino acids is indicated by the number of nucleotides. Example species from three different classes—*E. faecalis* (Bacilli), *C. tyrothricin* (Clostridia) and *A. geminatus* (Negativicutes)—from the phylum Bacillota are presented. **a**, Distribution of 5' endpoints relative to Ile codons in samples treated with MUP after 5 and 30 min compared with NT control. **b**, As in **a** but for Ala codons after CAM treatment. **c**, As in **a** but for start codons after DOX treatment. **d**, As in **a** but for the first codon of Lys (K)- and Arg (R)-enriched tripeptide motifs of the form (R/K) × (R/K) in samples treated with ERY.

**Extended Data Fig. 8:**
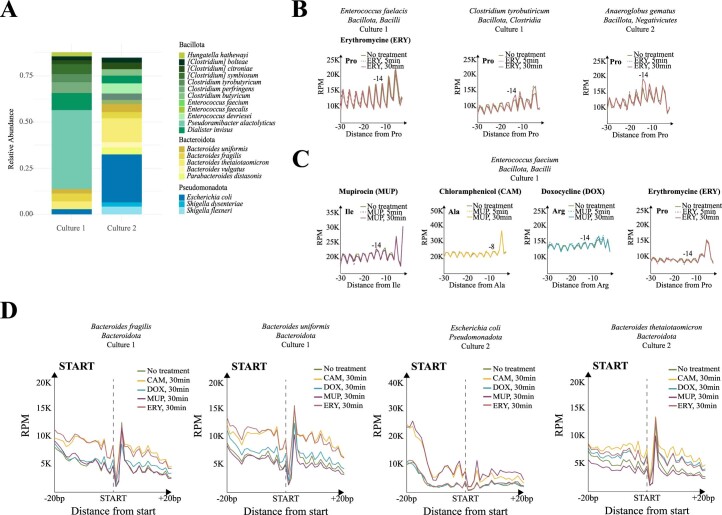
The composition of fecal cultures influences specie-specific response to antibiotic treatments. **A**, Relative composition of the used fecal cultures based on genomic DNA abundance. **B**, Line plots showing 5PSeq metagene abundance in respect to selected features illustrating species-specific differential response depending on the culture of origin. **C**, Example of the Bacillota *E. faecium* showing little response to the applied drug treatment. **D**, Bacteroidota species, lacking RNase J, also present clear alterations of the 5´P degradome profile in response to drug treatment.

We reasoned that 5P-seq might be capable of detecting species-specific posttranscriptional responses to drug treatments independently of changes in mRNA abundance. We analysed the metadegradome of complex faecal microbiome cultures rich in Bacillota and Bacteroidota in response to drugs targeting translation (Fig. 4 and Extended Data Fig. 8a). We used CAM, MUP, doxycycline (DOX, inhibition of translation elongation) and erythromycin (ERY, interference with aminoacyl translocation). First we focused on Bacillota (containing RNase J) where metadegradome sequencing provides higher resolution. As expected from our previous analyses in isolated species, the use of CAM and MUP led to context-specific stalls at Ile and Ala in Bacillota, such as in *Enterococcus faecalis* (class Bacilli), *Clostridium tyrobutiricum* (class Clostridia) and *Anaeroglobus geminatus* (class Negativicutes) (Fig. 4a,b). For DOX we observed a clear 5′P RNA accumulation –11 nt from the start codon, indicating protection of ribosomes paused at the level of initiation (Fig. 4c). For ERY we observed ribosome stalling associated with positively charged amino acids including Lys (K) and Arg (R). This specifically occurred for motifs (R/K) × (R/K) (Fig. 4d) and Pro (Extended Data Fig. 8b), in agreement with previous reports^43–46^. Effects were observed as early as 5 min after treatment, with maximal effects after 30 min (Fig. 4). This delayed response is consistent with a slower growth rate of faecal cultures and a lag in translational response. Interestingly, each drug had varying effects on bacterial species in the mixed cultures, suggesting that bacterium-specific physiological state and culture composition can modulate the ability of cells to respond to translation perturbation: for example, *Enterococcus faecium*^46^ did not respond to drugs in our experiments (Extended Data Fig. 8c).

In our microbiome experiments we also identified diversity in Bacillota ribosome stalling mechanisms. Species in classes Clostridia and Negativicutes—for example, *C. tyrobutiricum* and the opportunistic pathogen *A. geminatus*—have an additional protection signature 2 nt upstream of a ribosome stall (Fig. 4). This occurred with all the drugs tested and affected ribosomes stalled at either elongation or initiation. Additionally it was conserved across phylogenetically related species, suggesting that stalled ribosomes in those species interact with additional cofactors, or present alternative conformation, that alters in vivo ribosome protection patterns.

Finally we tested the effect of antibiotic treatments on the metadegradome profile of Bacteroidota and Pseudomonadota species lacking 5′–3′ exonuclease activity. Although we did not expect single-nucleotide information about differential ribosome stalling, antibiotic treatments led to an accumulation of 5′P sites around the start codon, as observed for CAM and ERY in *Bacteroides uniformis* and *Bacteroides fragilis*, for DOX in *Bacteroides thetaiotaomicron* and for MUP and CAM in *E. coli* (Extended Data Fig. 8d). In summary, we have shown that metadegradome sequencing can reveal species-specific information on posttranscriptional regulation in complex microbiome samples.

## Bacterial mRNA degradome atlas

**Fig. 5:**
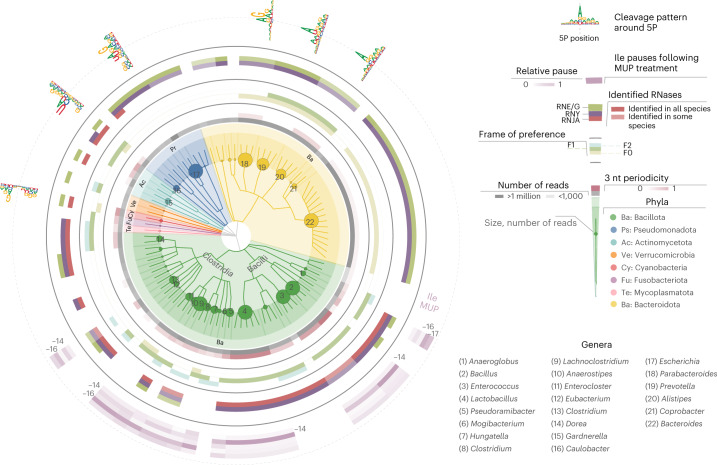
Co-translational mRNA decay is conserved across prokaryotes. Taxonomic tree of investigated species. Circles, from inside to outside, grey (number of assigned reads); red (strength of 3 nt periodicity), frame preference (F0, beige; F1, green; F2, light blue); identification of selected enzymes involved in RNA degradation at genus-level RNJA, RNY and RNE/G; ribonucleases from E/G protein family; accumulation of 5′P endpoints 13–17 nt before Ile codons after 30 min MUP treatment; cleavage motifs 4 nt around 5′P endpoints of selected species (skipping Bacillota with no cleavage preference (Fig. 1c)). Overall, 58 genera were present in samples from cultured bacteria and complex environments, including vaginal swabs, faeces, faecal cultures and compost. In complex samples, species with at least 1,000 reads in the coding regions (300 in the case of compost) were considered, and respective genera analysed (Methods).

To showcase the breadth of uses for our method, we compiled an atlas of mRNA decay across the bacterial tree of life (Fig. 5). We analysed 96 species across 58 genera (Fig. 5 and Supplementary Tables 5–7), identifying agreement between mRNA degradation patterns and taxonomic classification and the existence of 3 nt periodicity in most genera (Fig. 5, inner barplot in red). Genera containing RNase J, such as those in Bacillota (in green), have a clear 3 nt periodicity with a general preference for 5′P in frame F1 (for example, spp. *Lactobacillus*, *Bacillus* and *Megasphaera*). On the other hand, Bacteroidota that are more dependent on endonucleolytic cleavage, with RNase E and G family proteins and RNase Y, present 5′P sites frequently associated with the last nucleotide of the codon (F2; Fig. 5, coloured beige) in, for example, *Parabacteroides*, *Alistipes* and *Bacteroides*. Many species have more complex codon-associated 5′P patterns. For example, *Ureaplasma* (Mycoplasmatota) and *Akkermansia* (Verrucomicrobia) have 5′P sites in F1 and F2 while *Caulobacter* (Pseudomonadota) and *Prevotella* (Bacteroidota) are associated with F1 and F0. It is important to note that both 5′P preference and ribosome protection can be influenced by environmental conditions or drug treatments^19^ as well as by taxonomy. A few species for which we produced deep-sequencing coverage (Fig. 5, inner grey circle), such as *E. coli* (representative of class Gammaproteobacteria), do not show a clear preference for 5′P in respect to codon positions. Consistent with our previous analysis of sequence preference for 5′P sites (Fig. 1c), we observe that species with RNase J show little 5′P sequence-specific preference. Species more dependent on the endonucleolytic activity of RNase G often use –1 U/+1 A while those dependent on RNase Y present a clear enrichment for +1 A. To further dissect the interplay between RNA degradation machinery and ribosome position, we investigated the protection of stalled ribosomes at Ile codons following MUP treatment (outer purple circle, Fig. 5). Focusing on Bacillota, in addition to the expected protection size (that is, 14 nt, as in Fig. 2), we observed protections at –16 nt in the genus Clostridia indicating divergent mechanisms in response to stalled ribosomes across phylogenetically related species. These results show that global investigation of in vivo mRNA degradation, combined with translation perturbation, is a useful tool in the discovery of both currently uncharacterized mechanisms for translation regulation and species-specific response to antibiotic treatments.

To facilitate the use of our resource investigating the interplay between RNases and the translation process across bacteria, we provide an interactive browsable website for further exploration (http://metadegradome.pelechanolab.com).

## Discussion

We investigated mRNA degradation intermediates in isolated species and complex microbiomes using an optimized 5PSeq method and found that co-translational mRNA degradation is widespread among bacteria. In species that contain RNase J, 5′P mRNA decay intermediates provided ribosome protection information at single-nucleotide resolution. Our approach enables the study of in vivo codon- and gene-specific ribosome stalls without the need for drug treatment, subcellular fractionation or in vitro RNA degradation. We show that optimized 5PSeq can detect environmentally triggered translational regulation in multiple species, at both the codon- (Fig. 2) and gene-specific level (Supplementary Table 3). In species without 5'–3' exonuclease activity, perturbation of the translational process resulted in alterations of 5′P distribution along the genes. This suggests that, despite lacking a 5'–3' exonuclease able to toeprint the ribosome position at single-nucleotide resolution, changes in ribosome position also shape the co-translational mRNA degradome in those species. We exploited co-translational decay processes in bacteria to show that 5PSeq can detect rapid post-transcriptional responses (within 5 min) to perturbations of the translational process by drug treatment, independent of changes in mRNA abundance. Because co-translational mRNA decay is prevalent but distinctive among bacterial species, a 5PSeq signature can be applied to capture species-specific post-transcriptional responses to perturbations. We propose that adoption of our method potentially enables the study of environmental or chemical regulation of translation without the need for culturing or subcellular fractionation. Using our optimized method we show that ribosome position is key for shaping the boundaries and abundance of bacterial degradomes. We show that environmental changes leading to stress-specific defects in tRNA biosynthesis or processing^32^, or changes in amino acid availability^47^, alter the degradome in a predictable way in species containing RNase J. Additionally our work suggests that bacterial stringent response, in addition to controlling transcription and translation^35,48^, also alters the bacterial degradome. We also show that ribosome stalls and associated co-translational decay patterns are highly regulated across stress conditions and can be used to investigate gene-specific alterations independent of changes in mRNA abundance. For example, we observed clear changes in gene-specific 3 nt periodicity (FPI) for genes associated with translation and spore wall formation in *B. subtilis* (Extended Data Fig. 5). By dissecting the contribution of different RNases to the degradome in *B. subtilis*, we also identified gene-specific regulation of regulatory 5' UTRs and small regulatory RNAs (Supplementary Table 4) as well as their interaction with bacterial riboswitches^49^. This information will be useful for the future dissection of regulatory elements in mRNA leaders, and to facilitate the molecular dissection of their mechanism of action. Importantly, the metadegradome analysis performed in this manuscript is directly applicable to a wide variety of culturable and unculturable species where RNase abundance can be readily inferred (Supplementary Table 6), thus paving the way for the molecular dissection of RNA decay in non-model bacterial species.

Finally, we show that 5PSeq can be widely applied to complex clinical and environmental microbiomes to obtain species-specific metadegradome profiles. By eliminating the need for complex experimental protocols and bacterial culturing, researchers will be able to analyse ribosome protection phenotypes in uncultivable bacterial species, which are estimated to comprise more than half of human bacterial communities^50^. Our approach can detect translational responses even in species present at 0.01% abundance in a bacterial community and, by using the longer ribosome toeprints, it enables analysis of complex bacterial communities at single-species resolution. To demonstrate its utility and ability to complement current metagenomic approaches, we characterize ribosome dynamics in complex faecal cultures after antibiotic treatment. Our work shows that 5PSeq can readily identify rapid post-transcriptional responses to antibiotics targeting the translational process. This confirms our ability to investigate codon-specific changes in complex microbial samples and paves the way for the use of degradome signatures as molecular reporters for species-specific responses to drugs. We provide an atlas of bacterial mRNA degradation for 96 species spanning 58 genera. Our results highlight the diversity of mRNA degradation machinery across prokaryotes and demonstrate the utility of combining translation perturbation and the 5′P mRNA degradome. We expect that, by combining 5′P RNA sequencing (Supplementary Table 4) with a measure of the expressed RNases (Supplementary Table 7), in the future the study of the metadegradome will facilitate the mechanistic characterization of regulatory 5' leaders in less-well-characterized species.

In summary, our analysis of mRNA degradation across the bacterial tree of life reveals how the metadegradome provides valuable information regarding species-specific physiology including responses to drugs, intracellular amino acid availability and environmental responses. Metadegradome analyses will enable characterization of post-transcriptional regulation in microbiomes and shed light on the biology of microbial communities.

## Methods

### Bacterial growth

Overnight bacterial cultures (not exceeding optical density 1 (OD_600_ = 1) were used to dilute the main culture to a starting OD_600_ of 0.03–0.05. Cultures were harvested by centrifugation on reaching logarithmic phase (OD_600_ = 0.4–0.8) unless indicated otherwise. If not stated differently, bacterial cultures were grown at 37 °C with rotation using the recommended growth media. Specifically, *L. plantarum* (ATCC 8014) and *L. reuteri* (DSM 17938) were grown in MRS broth (Sigma-Aldrich). *L. plantarum* stress treatments were carried out as follows: stationary-phase cultures were grown for 27 h post inoculation and harvested at OD_600_ = ~4.5. To generate samples for untreated control, heat shock and low-nutrient, biological replicate cultures (40 ml) were grown to mid-log phase (OD_600_ = 0.3–0.6), split (10 ml for untreated control, 15 ml for heat shock and 15 ml for low-nutrient sample) and cells harvested by centrifugation. Untreated control pellets were flash-frozen immediately for RNA analysis. Before heat shock, cells were resuspended in prewarmed MRS broth and incubated in a thermomixer for 15 min at 60 °C. Low-nutrient cell pellets were washed thoroughly with 50 ml of 0.5× LB medium (Sigma), centrifuged and the supernatant completely removed. Cells were then resuspended in prewarmed 0.5× LB medium (Sigma) and harvested after a total incubation time of 15 min at 37 °C. *B. subtilis* (168trpC2) was cultured in 2× YT (1.6% (wt/vol) tryptone (Bacto), 1% (wt/vol) yeast extract (Bacto) and 0.5% (wt/vol) NaCl. For extended growth we collected samples at mid-log, 24 h, 48 h and 8 days post inoculation. Salt stress was performed by mixing equal volumes of 2 M NaCl with mid-log-phase-grown *B. subtilis* (OD_600_ = 0.5–0.6), followed by 10 min incubation at room temperature and harvesting by centrifugation. Biological replicate cultures of *L. plantarum*, *L. reuteri*, *E. coli* (Invitrogen, no. 18265-017) and *B. amyloliquefaciens* were grown in LB medium to log phase, then cultures were split to generate samples for untreated control and random fragmented control. CAM was added to mid-log-phase-grown *L. plantarum* and *L. reuteri* cultures at a final concentration of 100 µg ml^–1^, incubated for 5 min at 37 °C and subsequently harvested on ice containing an additional 100 µg ml^–1^ CAM^5^. MUP treatment (final concentration 65 µg ml^–1^) of mid-log *L. plantarum* and *L. reuteri* was carried out for 10 min at 37 °C following centrifugation and flash-freezing of the pellet. *C. crescentus* (strain NA1000) was grown in PYE medium containing 0.2% (wt/vol) peptone (Bacto) and 0.1% yeast extract (Bacto) at 30 °C to mid-log, followed by centrifugation and flash-freezing of the cell pellet. *Synechocystis* strain PCC6083 was cultured in BG11 growth medium at 30 °C at a light intensity of 30 µE and 1% atmospheric CO_2_ and harvested at mid-log phase^51^. Heat shock treatments for *E. coli*, *B. subtilis* wild-type and RNase knockout strains were carried out as follows: *E. coli* (MG1655) strain was grown in LB medium at 37 °C to late log phase (0.6–0.8); *B. subtilis* wild-type (168trpC2), *rnjA*, *rnjB* and *rny* deletion strains^41,52^ were cultured at 37 °C in LB (supplemented with 100 µg ml^–1^ spectinomycin for *rnjA* and 5 µg ml^–1^ kanamycin for *rnjB* and *rny* deletion strains) to log growth phase (0.2–0.3). The culture was split in two, the cell pellet collected by centrifugation and the untreated control sample flash-frozen immediately. Heat shock was carried out by resuspending cells in 65 °C prewarmed LB medium followed by 10 min incubation at 65 °C. Samples were harvested by centrifugation and shock-frozen in an ethanol/dry ice bath for RNA analysis.

For antibiotic treatment of *E. coli*, strain MG1655 was grown in LB at 37 °C to exponential phase and cultures divided in three for sampling of untreated control, MUP (65 µg ml^–1^) and CAM (100 µg ml^–1^). Cultures were incubated with antibiotics for 10 min at 37 °C, harvested by centrifugation and shock-frozen in an ethanol/dry ice bath for RNA analysis.

For expression of RNase J in *E. coli*, the top ten cells were transformed with plasmid containing the RNase JA coding sequence from *S. pyogenes* (plasmid pEC622:pEC85 with rnjA promoter + rnjA (expressing RNase J1 for complementation in *S. pyogenes* (pEC85 replicates in *E. coli*), a gift from E. Charpentier). Strains *A. finegoldii* (DSM 17242), *P. copri* (DSM 18205), *P. merdae* (DSM 19495) and *P. timonesis* (DSM 22865) were anaerobically cultured in GAM broth (HyServe) for 24 h. Where indicated, species were treated with either MUP (65 µg ml^–1^) or CAM (100 µg ml^–1^) for 10 min. After incubation, 1 ml aliquots were removed and cells catalytically inactivated by the addition of RNAprotect Bacteria Reagent (Qiagen) and harvested by centrifugation.

### Gut microbiome samples

To monitor the effects of drugs on the dynamics of the metadegradome, we screened two anaerobically cultivated human gut microbiomes against four drugs over time. Gut microbiomes from healthy patients were collected and resuspended in 40% glycerol and 500 µl aliquots were stored at –80 °C. One aliquot was used to inoculate 10 ml of starter culture maintained in MCDA broth for 24 h^53^ in the absence of oxygen and in the presence of 2.5–3.0% hydrogen at 37 °C. To ensure that the microbial community was in a metabolically active growth phase, we diluted the starter cultures with fresh MCDA medium at a 1:10 ratio 24 h before starting the experiment, in a total volume of 30 ml, under the anaerobic conditions given above but without shaking. For antibiotic treatment, 6 ml of faecal culture was transferred into a 15 ml Falcon tube either without antibiotics or supplemented with either CAM (80 µg ml^–1^), MUP (650 µg ml^–1^), DOX (5 µg ml^–1^) or ERY (5 µg ml^–1^). After incubation for 5 min, 30 min or 48 h, 1 ml aliquots were removed and growing cells catalytically inactivated by the addition of RNAprotect Bacteria Reagent (Qiagen) (*t*_0_ before treatment and at 5 min, 30 min and 48 h post treatment) and harvested by centrifugation. The number of replicates for each sample is listed in Supplementary Table 2.

### RNA extraction

Extraction of RNA (if not stated otherwise) was performed as described in ref. ^54^, with minor modifications. In brief, cell pellets were resuspended in equal volumes of LET (25 mM Tris pH 8.0, 100 mM LiCl, 20 mM EDTA) and water-saturated phenol pH 6.6 (Thermo Fisher). Cells were lysed with acid-washed glass beads (Sigma-Aldrich) by vortexing for 3 min in MultiMixer. Following the addition of equal volumes of phenol/chloroform/isoamyl alcohol pH 4.5 (25:24:1) and nuclease-free water, lysis was extended by an additional 2 min of vortexing followed by centrifugation. The resulting aqueous phase was purified in two steps using phenol/chloroform/isoamyl alcohol (25:24:1) followed by chloroform. Following centrifugation, the clean aqueous phase was precipitated with sodium acetate-ethanol. For *Lactobacillus* mixtures (Fig. 3a), microbial RNA extracted from *L. plantarum* (untreated control and MUP treated) were mixed before the RNA ligation step of the 5P-seq library protocol at different ratios with RNA extracted from *L. reuteri* (untreated). Technical replicates of the Microbial Community Standard (Zymobiomics, no. D6300, lot no. ZRC 190633), consisting of eight deactivated bacterial strains, were generated by extraction of RNA from 75–125 µl of thawed cell suspension. Vaginal swab samples were mechanically lysed using beads in 1,000 µl of DNA/RNA shield (ZymoResearch) and lysate was stored at –80 °C for 2 months before use. Lysate was thawed and 250 µl used to extract microbial RNA. Faeces from a healthy donor were collected and transported in 40% glycerol. Technical replicates of RNA were extracted on the day stated above, with minor modifications listed as follows. In brief, 500 µl of faeces/glycerol suspension was mixed with an equal volume of LET buffer containing SDS (25 mM Tris pH 8.0, 100 mM LiCl, 20 mM EDTA, 10% SDS) and water-saturated phenol pH 6.6 (Thermo Fisher). Lysis was performed by vortexing with carbide beads; the duration was extended to 10 min after the addition of equal volumes of phenol/chloroform/isoamyl alcohol pH 4.5 (25:24:1) and nuclease-free water. All subsequent steps were performed as described above. RNA from the cultivated gut microbiome time-course experiment was extracted as stated, with modifications to the LET buffer containing SDS (25 mM Tris pH 8.0, 100 mM LiCl, 20 mM EDTA, 10% SDS). Compost RNA was extracted with 2 g of starting material (from Sundbyberg, Sweden) using the RNeasy PowerSoil Total RNA Kit (Qiagen) as recommended in the manufacturer’s guidelines. RNA quality was assessed by loading of either 1 µg of total RNA on 1.2% agarose gel or 12 ng of total RNA on a BioAnalyzer using an RNA Nano 6000 chip (Agilent Technologies). Before 5P-seq library preparation, RNA was quantified using the Qubit RNA BR (Broad-Range) kit (Thermo Fisher Scientific) according to the manufacturer’s guidelines.

### DNA extraction

Purification of DNA was carried out by lysing cells from the gut microbial community with glass beads (Sigma), combined with phenol/chloroform/isoamyl alcohol extraction and ethanol precipitation. The workflow protocol was identical to the RNA extraction procedure described above but with the substitution of LET (25 mM Tris pH 8.0, 100 mM LiCl, 20 mM EDTA, 10% SDS) with 10 mM Tris-HCl pH 8.0 and the substitution of phenol/chloroform/isoamyl alcohol pH 4.5 with UltraPure phenol/chloroform/isoamyl alcohol pH 8.0 (Invitrogen)

### Polyribosome fractionation

Polyribosme fractionation was performed as previously described^55^, with minor modifications. In brief, *B. subtilis* (168trpC2) was cultured in LB medium to mid-log phase at 37 °C and harvested on ice containing 100 µg ml^–1^ CAM, with 5 min centrifugation. The resulting pellet was lysed in 1 × TN (50 mM Tris/HCl pH 7.4, 150 mM NaCl, 1 mM DTT, 100 μg ml^–1^ CAM and a complete EDTA-free protease inhibitor tablet) using glass beads with vortexing for 2 min, followed by 5 min incubation on ice. Lysis and incubation were repeated twice. Cell debris was cleared by centrifugation at 1,500*g* for 5 min at 4 °C and the supernatant loaded onto a 15–50% sucrose gradient with an 80% cushion. After ultracentrifugation at 36,000 rpm for 90 min, Abs_254_ was monitored and fractionated. Subsequently, RNA was extracted from sucrose fractions by additionof equal volumes of phenol/chloroform/isoamyl alcohol pH 4.5 (25:24:1) and nuclease-free water, followed by 2 min of vortexing and centrifugation. The aqueous phase was further cleaned by the addition of chloroform, vortexing and centrifugation and the resulting aqueous phase sodium acetate/ethanol precipitated.

### Preparation of 5P-seq libraries

Our 5P-seq libraries were prepared as previously described^5,41^, with minor modifications, using 150–9,000 ng of total RNA as input. To prepare random fragmented samples (negative controls), ribosomal RNA was depleted from DNA-free RNA and subsequent fragmentation by incubation for 5 min at 80 °C in fragmentation buffer (40 mM Tris acetate pH 8.1, 100 mM KOAc, 30 mM MgOAc). The reaction was purified using two volumes of RNACleanXP beads (Beckman Coulter) as recommended by the manufacturer. Free 5' OH sites were rephosphorylated using 5 U of T4 polynucleotide kinase (NEB) and incubated at 37 °C for 60 min, as recommended by the manufacturer. Rephosphorylated fragmented RNA was purified using phenol/chloroform/isoamyl alcohol (24:25:1) followed by sodium acetate/ethanol precipitation. From this step forward, procedures for random fragmented and standard 5P-seq library preparation were merged^41^. RNA was ligated to either the rP5_RND or rP5_RNA oligo (Supplementary Table 1) containing unique molecular identifiers. Ribosomal RNA was depleted using the Ribozero rRNA removal kit (Illumina), which is suitable for bacteria, yeasts and human samples. The rRNA-depleted sample was purified using 1.8 volumes of Ampure beads (Abcam) and fragmented by heat (80 °C) for 5 min in 5× fragmentation buffer (200 mM Tris acetate pH 8.1, 500 mM KOAc,150 mM MgOAc). Subsequent samples were reverse transcribed using random hexamers for priming. The resulting complementary DNA was bound to streptavidin beads (M-280), subjected to enzymatic reactions of DNA end repair and filling in of adenine to the 5' protruding ends of DNA fragments using Klenow Fragment (NEB). The common adaptor (P7-MPX) was ligated and 5P-seq libraries were amplified by PCR (15–17 cycles), purified using 1.8 volumes of Ampure beads (Abcam) and quantified with Qubit (Thermo Fisher). Library size was estimated from bioanalyser traces. 5P-seq libraries were pooled by mixing equal amounts of each sample, followed by enrichment of 300–500-nt fragments.

During the work described in this manuscript, our laboratory developed a simplified high-throughput 5P-seq strategy that was applied to a subset of samples, as detailed in Supplementary Table 2. Libraries compiled by this strategy were generated as recently described^15,56^. In brief, DNA-free RNA was ligated with RNA oligos containing unique molecular identifiers. Ligated RNA was reverse transcribed by priming with oligos containing a random hexamer and an Illumina-compatible region. RNA was eliminated by the addition of NaOH. Ribosomal RNA was depleted by the addition of in-house rRNA DNA oligo depletion mixes (Supplementary Table 1) to cDNA and performing duplex-specific nuclease (DSN, Evrogen) treatment. rRNA-depleted cDNA was PCR amplified (15–17 cycles). Depletion of rRNA with Ribozero Illumina (for bacteria and yeasts) was done after the single-stranded RNA ligation step. Ribosomal-depleted RNA was purified and reverse transcribed using the oligos mentioned above and then amplified by PCR. Libraries were quantified by fluorescence (Qubit, Thermo Fisher), their size estimated using an Agilent Bioanalyser and sequenced using either a NextSeq500 or NextSeq2000 Illumina sequencer.

### Metagenomic library preparation

DNA libraries were prepared from time points of untreated (*t*_0_) and 48 h (*t*_48h_)-treated faecal cultures according to the manufacturer’s guidelines (ThruPlex DNA-Seq Kit, Takara Bio). In brief, DNA was sheared using an ME220 focused ultrasonicator (Covaris) to an average size of 350 nt (microtube AFA Fiber crimp-Cap, PN 520053). Sheared DNA (30 ng) was used to implement a DNA end repair reaction, followed by library synthesis and PCR amplification (ten cycles) with primers NEBi5 and PE2. DNA libraries were purified using Ampure XP, quantified by fluorescence (Qubit, Thermo Fisher) and sequenced using a NextSeq2000 Illumina sequencer.

### Sequence data preprocessing and mapping

Demultiplexing and fastq generation of sequencing bcl image files was performed using bcl2fastq (v.2) with default options. Adaptor and quality trimming was performed with the bbduk programme of the BBTools suite (https://sourceforge.net/projects/bbmap/), with options (qtrim=r, ktrim=r, hdist=3, hdist2 = 2, K = 20, mink=14, trimq=16, minlen=30, maq=16), using the BBTools default adaptor set and polyG or polyA sequences for short reads. To reduce computational time, reads with both identical unique molecular identifier (UMI) and insert sequence were deduplicated before mapping using the deduping programme of the BBTools suite with default parameters. UMI sequences found in the first eight bases of each read were extracted using UMI-tools (v.1) with default options (using --bc-pattern NNNNNNNN).

Bacterial genomes were downloaded on 21 March 2019 from the National Center for Biotechnology Information Assembly database (https://www.ncbi.nlm.nih.gov/assembly/) with the following search terms: “bacteria”[Filter] AND (latest[filter] AND (“representative genome”[filter] OR “reference genome”[filter]) AND (all[filter] NOT “derived from surveillance project”[filter] AND all[filter] NOT anomalous[filter])). The list was further filtered to include only one strain per species, giving priority to genomes marked as “reference”. The resulting 5,804 genomes (Supplementary Table 4) were used to build the reference index. The index was built with the bbmap programme of the BBTools suite, with default options (and *k* = 10). Besides the reference index containing the 5,804 bacterial genomes, separate indices were built for individually cultured species and genus-level groups. Genomes for the latter groups were chosen from the initial set of 5,804 genomes. Alignment was performed with the bbmap programme of the BBTools suite, with the parameters (32 bit=t -da -eoom k = 11 strictmaxindel=10 intronlen=0 t = 16 trd=t minid=0.94 nzo=t). Alignment files were sorted and indexed with SAMtools^57^. Deduplication based on UMIs was then performed with UMI-tools (v.1)^58^, with the options (--soft-clip-threshold 1 --edit-distance 2 --method unique). The BAM files were then processed to enumerate the number of reads per species. We used a prestored dictionary of chromosome and species names and a custom script to perform counts in each species. The distribution of counts between genes coding for rRNA, tRNA, mRNA and other RNA types was computed with bedtools (https://bedtools.readthedocs.io/en/latest/). Counts at mRNA coding genes were used to select the top species in complex samples, as described below.

Individually cultured species were directly mapped to their reference indices. All Zymobiomics mixtures and vaginal, faecal and compost microbiome samples were aligned to the bacterial reference index that included the 5,804 species. We chose species with at least 1,000 reads in the coding regions in all the samples except for compost, where we relaxed the selection to 300 reads because there were fewer species with high counts. In total, 83 bacterial species with specified coverage belonging to 46 genera were identified from all the samples. Reference indices were built for these 46 genera (species were chosen from the preselected list of 5,804), and all complex faecal and compost samples were separately aligned to those references.

### 5PSeq and ribosome dynamics analysis

Deduplicated alignment files, along with genome sequence and annotation files, were provided as input to our recently developed fivepseq package^14^ for analysis and visualization of the 5' endpoint distribution of reads with default options applied. Fivepseq provides information regarding the presence of 3 nt periodicity (FFT), distribution of 5' counts relative to CDS start/stop or to nucleotides within each codon (translational frames), and codon and amino acid-specific protection patterns. Because fivepseq analyses only one genome per run, alignment files for complex samples were used as input for fivepseq for each genome separately. For genus-level analysis, sequence and annotation files for individual species were concatenated into one. Finally we compiled an online resource with interactive browsing of reports produced from the untreated bacteria with high coverage, at http://metadegradome.pelechanolab.com.

To generate a ribosome protection phenotype we took the sum of counts positioned 30 to 1 nt upstream of each amino acid and concatenated per-amino acid-scaled counts to obtain a vector describing ribosome protection in each sample. These vectors were used as input for PCA performed with the *prcomp* function of the R package stats (v.3.6.1). PCA plots were generated with the autoplotly package (v.0.1.2) in R.

### Analysis of degradome fragments with respect to genomic features

*Bacillus subtilis* annotations for transcriptional start sites (TSS) and UTRs were obtained from BSGatlas^59^. *E. coli* annotations for TSS and UTRs were obtained from RegulonDB^60^. 5′P reads were assigned to a given TSS if overlapping with that TSS within a ±5 base pair window. For reads distribution (Extended Data Fig. 6a) and heat maps (Extended Data Fig. 6e,f), replicates were pooled and either raw or library size-normalized read counts were averaged over replicates. The strand-specific coverage of 5′P reads was computed and represented as heat maps using deepTools (v.3.3.2)^61^. For differential analysis of 5′P reads in different genic annotation features, those in each genic annotation feature were enumerated using featureCounts from the R package Rsubread (v.2.6.4)^62,63^. Differential analyses were performed using edgeR (v.3.34.1)^63^.

### Cleavage motifs

The 5′P cleavage motifs were computed from the sequence composition of transcripts, involving region 4 nucleotides upstream and downstream of the 5' mapping positions of all reads. If multiple reads mapped to the same position, we considered the base composition of that region multiple times. Using the obtained base frequencies, we then proceeded to producing sequence logos with the R package ggseqlogo^64^, using Shannon entropy (bits) to compute the contribution of each nucleotide at each position.

### Taxonomic analysis

Taxonomic trees were generated with the graphlan tool (v.0.9.7) (https://huttenhower.sph.harvard.edu/graphlan). Taxonomic lineage information for all 84 bacterial species identified in our samples was downloaded from the NCBI Taxonomy database with the efetch programme from NCBI e-utilities. Trees were annotated with information on library size, 3 nt periodicity, preferred ribosome protection frame and the presence of enzyme annotations for each genus (Supplementary Data 1). Library size equalled the maximum number of mRNA reads per species per sample, in the range 0–1 (≥1 million) reads. The 3 nt periodicity was computed taking into account the absolute value of FFT signal for the 3 nt periodicity wave and the preference for ribosome protection frame, as computed by the fivepseq package. For FFT, the maximum number of signals for transcripts aligned either at the start or the end was taken. The preference for ribosome protection frame was assessed based on the value of FPI, computed by the fivepseq package as 2 × F/(∑_*1*_F_*1*_ − F_*i*_), for each frame F_*i*_. The frame with maximum absolute FPI value was regarded as (mis)preferred, and the significance of this preference was assessed based on *t*-test *P* values comparing counts in the given frame with the other two combined (FPI and *P* values are found in the frame_stats.txt file of the fivepseq output). A positive FPI value means that one of the nucleotides in each codon on average has higher counts (is preferred) while a negative value means that one of the nucleotides on average has low counts (is misprefered) and the other two nucleotides receive higher counts: for example, if F_1_ is preferred it will have a positive FPI value and will be highlighted in the tree as a single preferred frame of protection while if, say F_2_ is (mis)preferred (has a negative FPI value), the tree will highlight F0 and F1 as the frames of preference. Both FFT and FPI values were in the range 0–1, and the maximum of the two values was taken to describe the strength of 3 nt periodicity. Enzyme annotations were obtained from the EggNOG database (v.5.0)^65^ The presence of each enzyme in each genus was counted as a number between 0 and 1, depending on the fraction of species within the genus annotated with the enzyme. The tree highlights these values with corresponding opacity.

### Functional analysis

Functional analysis was performed on lists of genes with either differential abundance or differential frame protection between stress and control conditions. The FPI for each transcript was computed as described above. The fold change of FPIs was computed as the difference in mean FPI between the stress/mutant condition and the untreated/wild-type control for each transcript. Differential abundance of 5' endpoint counts for each transcript was computed as the expression log_2_(fold change) with the DESeq2 R package^66^ using adaptive Student’s *t*-test prior shrinkage estimation (apeglm).

Gene set enrichment analysis was performed with the R package WebGestaltR^67^ based on either fold change differential expression or FPI values. GMT formatted files for each species were obtained by modification of Uniprot protein annotations and used as enrichment databases. Annotation GFF files for each species were taken as the basis for generation of the reference gene lists. The significance of *P* values was computed with a hypergeometic test, and FDR used for multiple test correction.

### Ethical oversight

Collection and processing of sequenced vaginal samples was granted by the Regional Ethical Review Board in Stockholm (no. 2017/725-31). Ethical approval for faecal samples and cultures was waived by the review board because only deidentified samples from healthy donors were used and no samples were stored in a biobank. Informed consent was obtained from all donors before sample collection.

### Reporting summary

Further information on research design is available in the Nature Portfolio Reporting Summary linked to this article.

## Supplementary information

Supplementary Information Supplementary Figs. 1–8, descriptions of Tables 1–7 and uncropped scans of gels.

Reporting Summary

Supplementary Data 1–7 Table 1: oligonucleotides used in this study. Table 2: summary of samples and libraries analysed in this study. Table 3: log_2_(fold change) values of the relative abundance of reads undergoing degradation and FPI values for each gene and Gene Ontology-based gene set enrichment analysis in stress versus untreated conditions for *B. subtilis* (subsp. subtilis 168trpC2)*, E. coli* (strain MG1655) and *L. plantarum*. Enrichment *P* values were computed with a hypergeometric test, and multiple test adjustment performed with FDR and the R package WebGestaltR. Table 4: effect of RNase knockout on the abundance of RNA originating from different genomic features in *B. subtilis*. The table includes log_2_(fold change) for UTRs, TSS, coding regions and miscellaneous RNAs and tRNAs. Table 5: list of 5,804 prokaryotic genome assemblies retrieved from NCBI and used for alignment. Table 6: list of species with relatively high coverage identified in all studied samples. Table 7: estimated expression of RNases using 5P–seq across the studied samples, in RPM.

## Data Availability

Sequencing data are deposited with GEO under accession no. GSE153497. All output files of fivepseq generated in this study are deposited in the SciLifeLab Data Repository: 10.17044/scilifelab.22284709. Coverage tracks for vaginal samples are deposited at 10.17044/scilifelab.22305991. Source data are deposited in Figshare at 10.17044/scilifelab.22305955.

## Code availability

The code release for the fivepseq v.1.2.0 programme is available at https://github.com/lilit-nersisyan/fivepseq/releases/tag/v1.2.0. The remaining scripts and source data files used to generate the figures are deposited with GitHub at https://github.com/lilit-nersisyan/Atlas-of-mRNA-translation-and-decay-for-bacteria/, release v.1.0 (10.17044/scilifelab.22305955).
