# Supplementary material for: Atlas of mRNA translation and decay for bacteria
Source: Nat Microbiol. 2023 May 22;8(6):1123–36. doi: 10.1038/s41564-023-01393-z (PMC10234816; doi:10.1038/s41564-023-01393-z)
Supplement: Supplementary file 1 — Supplementary Figs. 1–8, descriptions of Tables 1–7 and uncropped scans of gels. [file 41564_2023_1393_MOESM1_ESM.pdf]

nature microbiology

Article

https://doi.org/10.1038/s41564-023-01393-z

Atlas of mRNA translation and decay for bacteria

In the format provided by the authors and unedited

nature microbiology

Article

https://doi.org/10.1038/s41564-023-01393-z

Atlas of mRNA translation and decay for bacteria

In the format provided by the authors and unedited

Supplementary Information

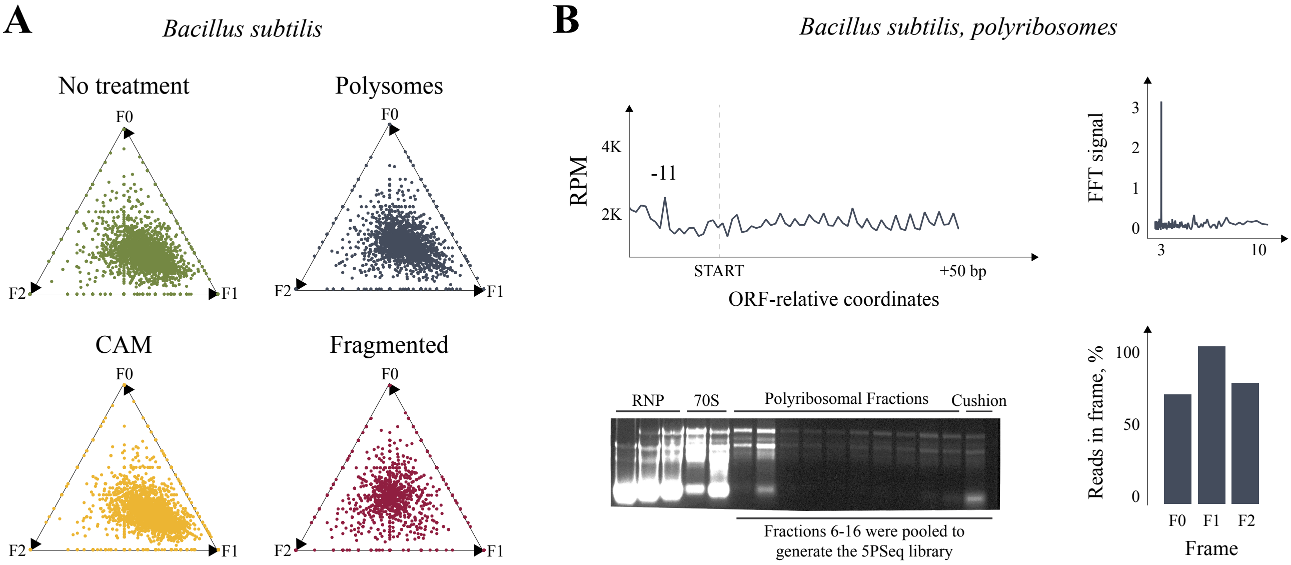

Extended Data Fig. 1. 5'P 3-nt periodicity is associated with co-translation mRNA decay. A, Gene-specific 3-nt protection for *B. subtilis* as reported by *fivepseq* [14]. Each point corresponds to a gene and the proximity to the triangle boundaries (F0, F1, or F2) their preferential protection frame. NT control cells are shown in green, polyribosome-associated mRNA degradation intermediates in blue, CAM treated cells in yellow, and randomly fragmented in red. **B**, 5PSeq metagene analysis of *B. subtilis* after polyribosome fraction isolation (as in Fig.1).

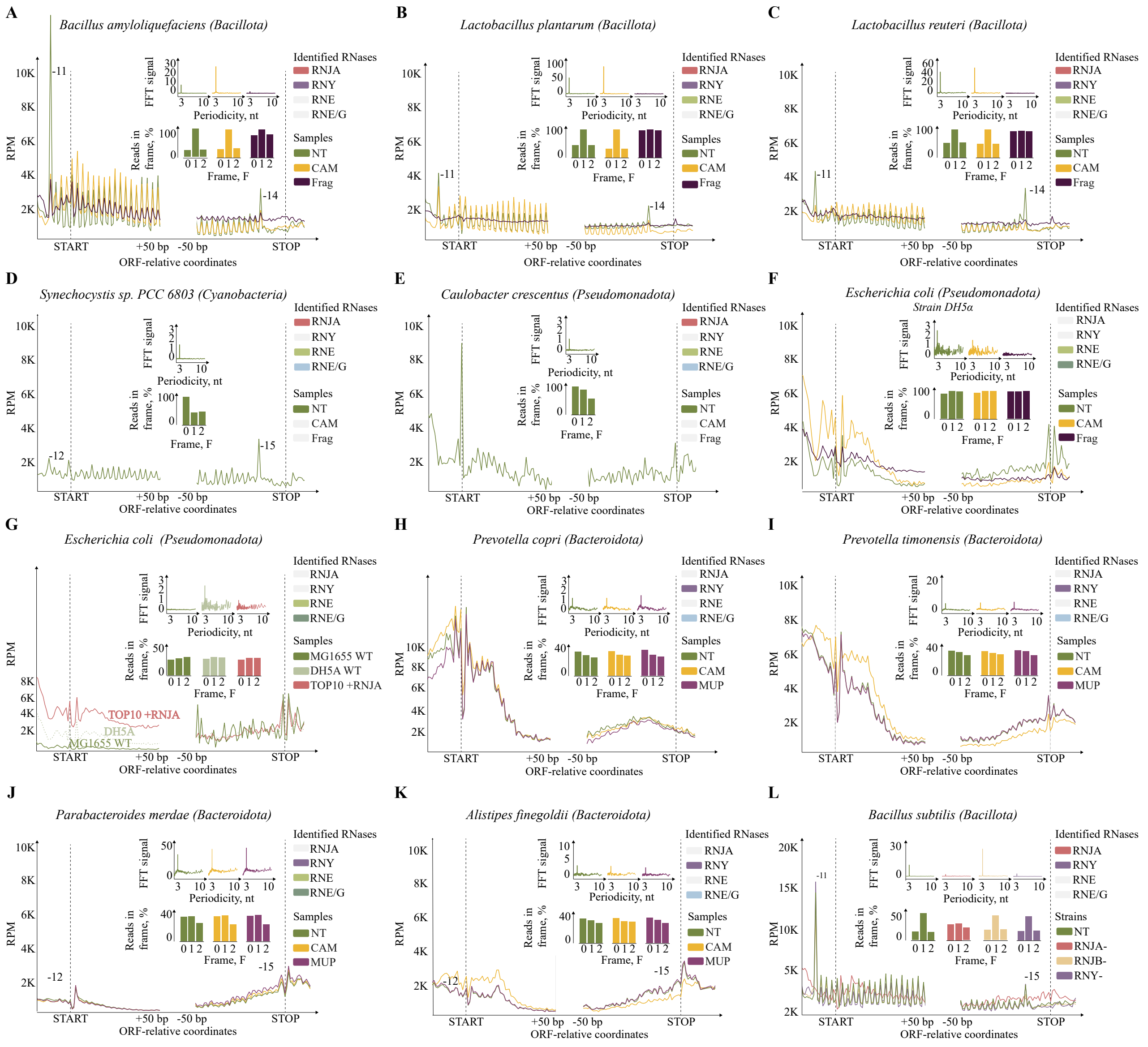

Extended Data Fig. 2. Ribosome-associated 3-nt periodicity can be found in multiple bacterial species. A-L, Metagene analysis for multiple species displaying metagene 5PSeq protection, Fast Fourier Transform (FFT), relative frame protection, and identified RNases (as in Fig.1).

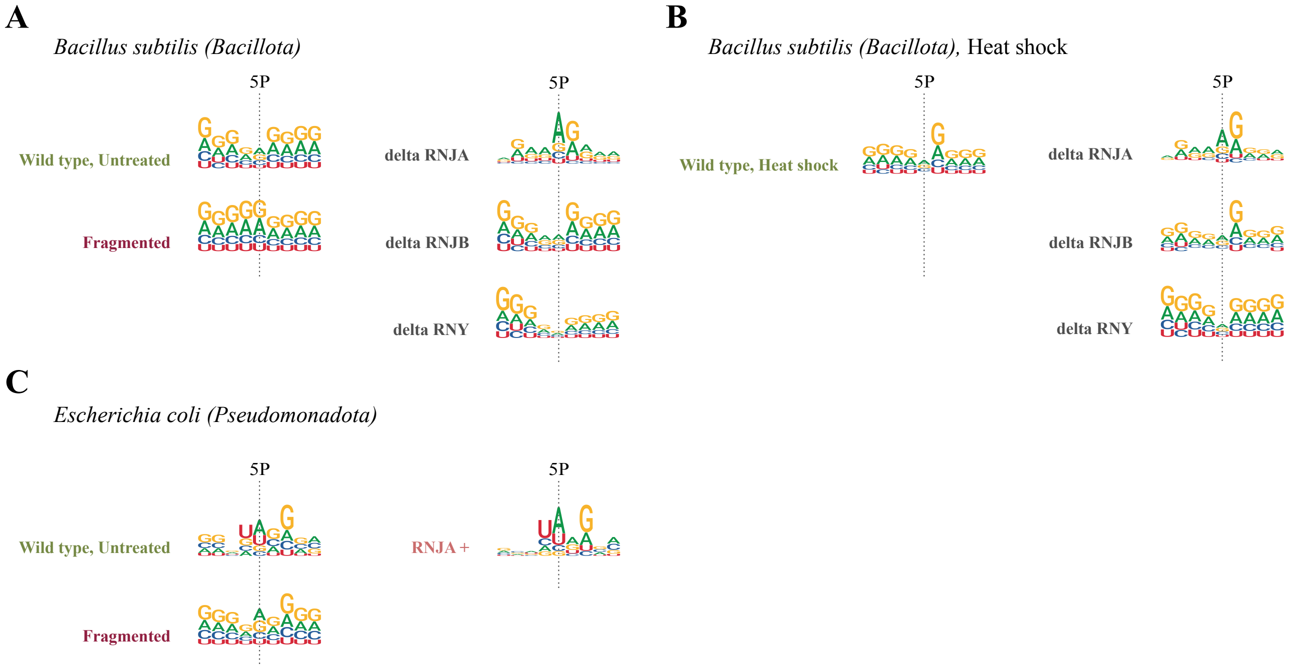

Extended Data Fig. 3. Cleavage motif preference at 5´P sites +/- 4 bases. A, NT and fragmented controls of *B. subtilis* wild type strains (168 trpC2), as well as rnjA, rnjB knock-out strains. **B** Wild type and RNase mutant *B. subtilis* strains under heat shock. **C**. NT control and fragmented samples of *E. coli Dh5*α strain and the TOP10 with heterologous expression of *S. pyogenes* RNJA. Cleavage motif preference was computed with Shannon entropy-based measure of nucleotide contribution (as in Fig. 1C).

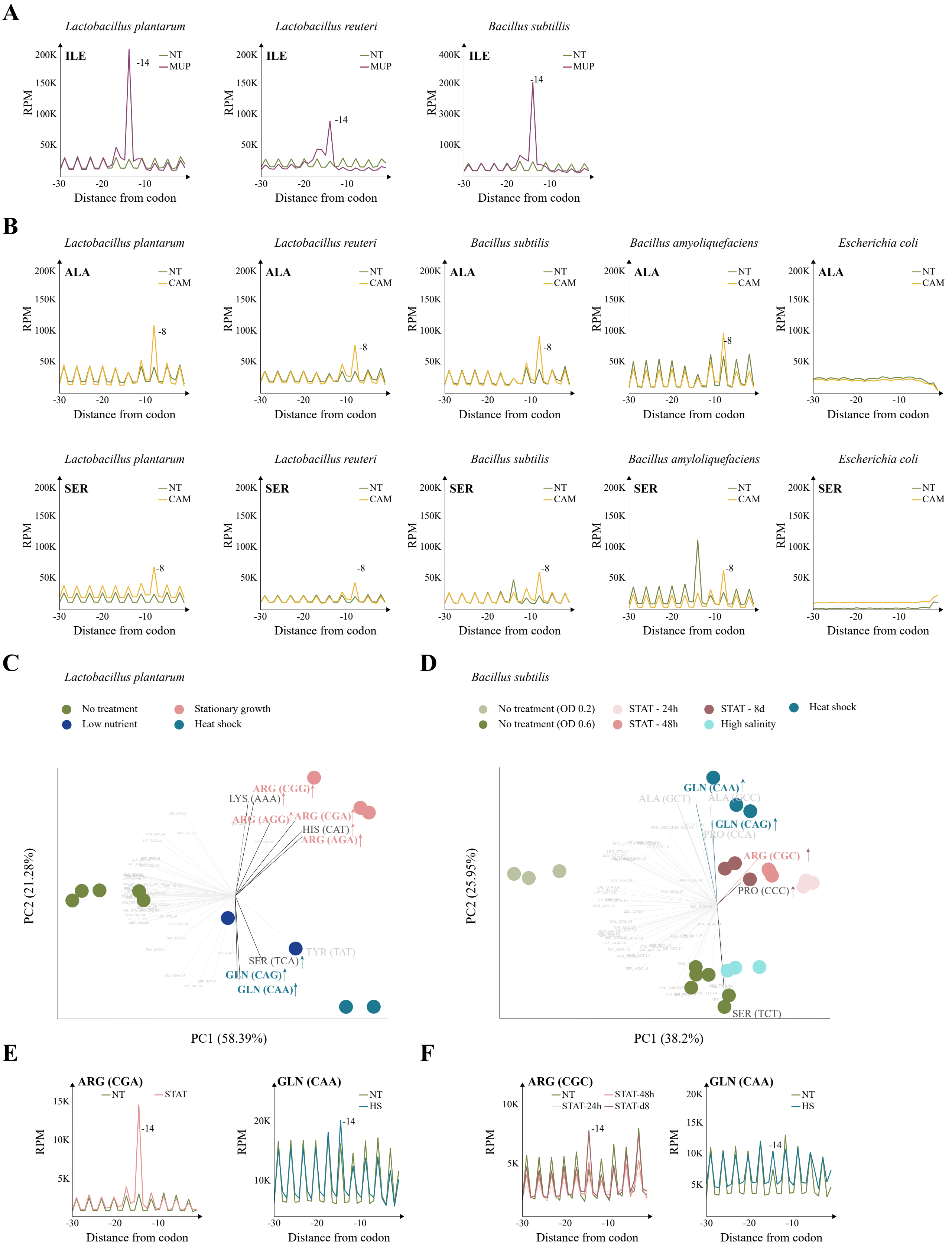

Extended Data Fig. 4. Species- and codon-specific ribosome pauses in response to stress or antibiotic treatment. Line plots showing amino acid-specific ribosome pauses as measured by 5PSeq. **A**, Isoleucine (Ile) pause (-14 nt) comparing NTand MUP treatment in *L. plantarum*, *L. reuteri* and *B. subtilis*. **B**, relative context-specific Alanine (Ala) and Serine (Ser) ribosome pause (-8 nt) as measured by 5PSeq for multiple species in response to CAM. Only in *E. coli*, which lacks RNase J, the 5´P degradome profile does not provide single-nucleotide resolution information of the ribosome stall. **C-D,** Principal component analysis plots based on 5P counts 14 nt upstream

from each codon for *L. plantarum* and *B. subtilis* in stress conditions. The NT control samples of *B. subtilis* have been collected at different growths phases (OD_{600} 0.2-0.3 and 0.6-0.8 respectively). The contribution of each codon is shown with gray loading vectors. The features with the longest loadings corresponding to those with highest absolute weight in the PC1 and PC2 are highlighted. The up arrows in labels indicate that the 5P counts increase in stress conditions for the codon. Codons of common amino acids between *L. plantarum* and *B. subtilis* are highlighted with stress-specific colors. **E-F**, relative Arg and Gln pauses (-14 nt) in stationary phase growth for *L. plantarum* (27 hours) and *B. subtilis* (8 days).

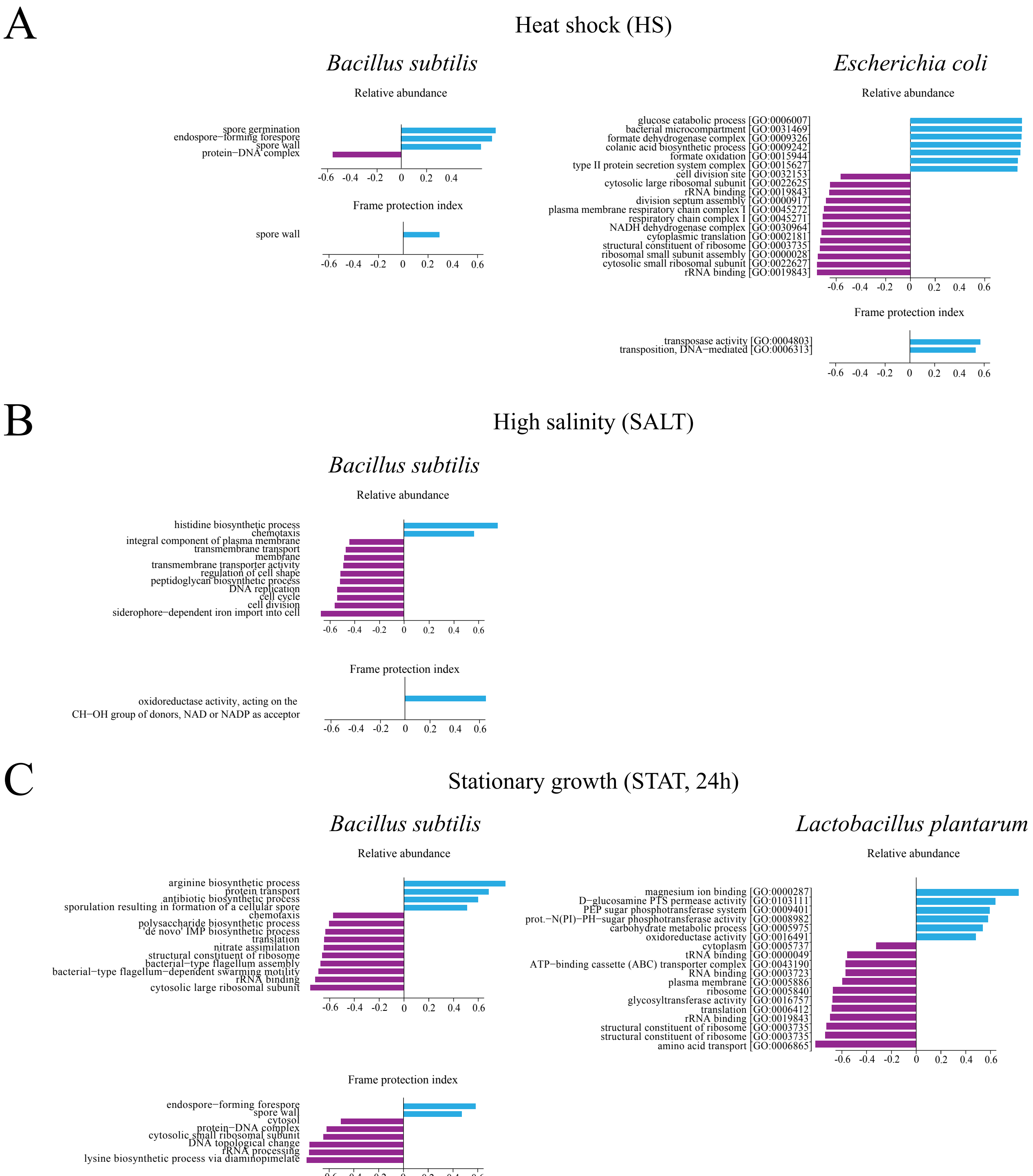

Figure S5. Gene set enrichment analysis of Gene Ontology functional sets in stress conditions. Either the log2 fold change of the reads undergoing degradation in stress compared to untreated samples (relative abundance) or the log2 fold change of the frame protection index (FPI) is considered when performing the enrichment analysis. Only results with false discovery rate of <0.05 are shown. **A.** *B. subtilis* and *E. coli* in heat shock. **B.** *B. subtilis* in high salinity. **C.** *B. subtilis and L. plantarum* in stationary growth.

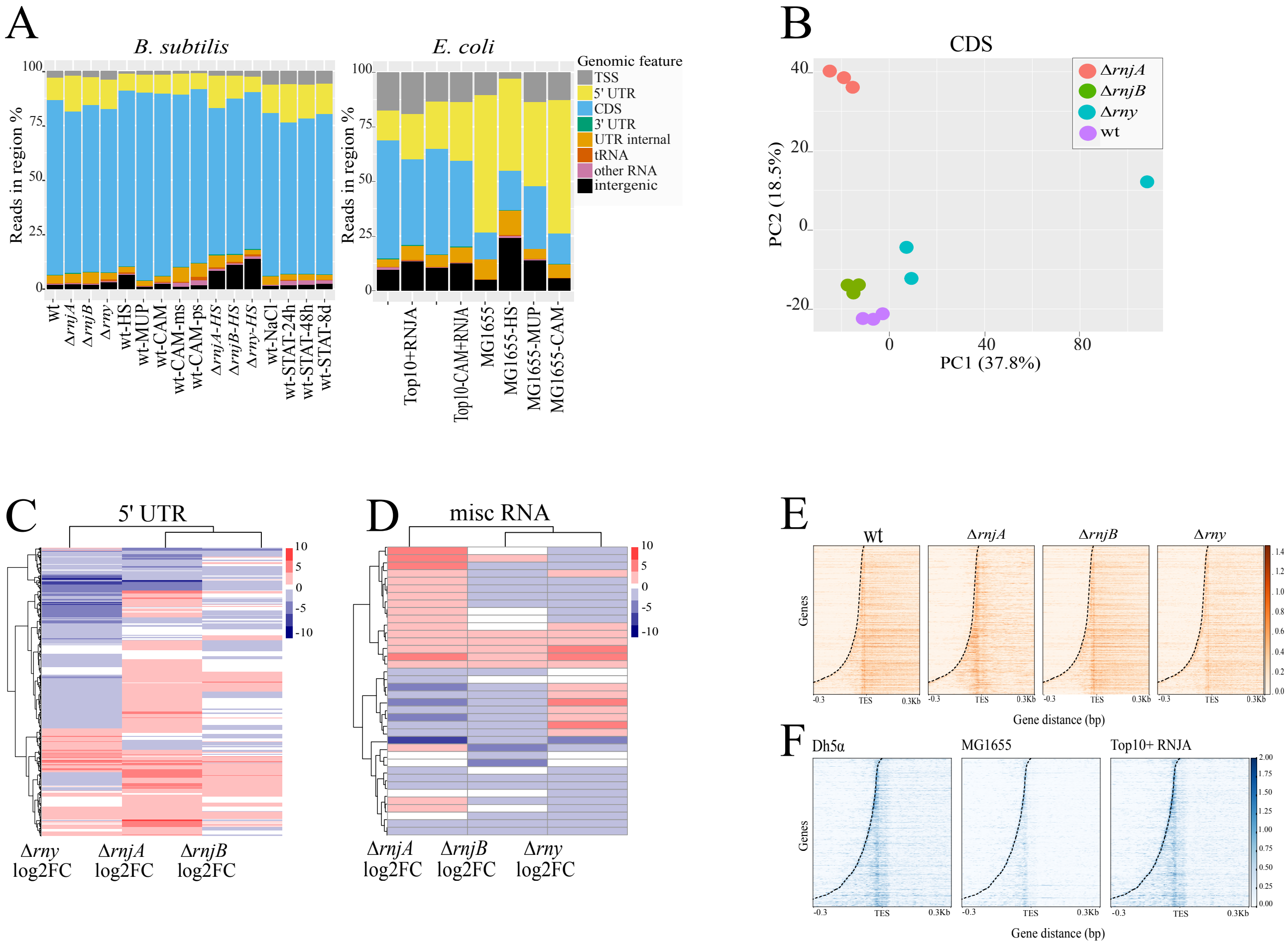

Extended Data Fig. 6. Analysis of degradome fragment in respect to genomic features. A, Barplots showing the percentage (%) (Y-axis) of 5' P reads overlapping a given genic annotation feature for *B. subtilis* (left) and *E. coli* strains (right panel) under normal and perturbed conditions. Contaminant rRNA reads were excluded from the analysis. **B**, Principal component analysis based on library size normalized 5' P reads on the natural logarithmic scale of *B. subtilis* control and different RNase knockdowns. Axes represent principal components 1 and 2. **C**, Heat map showing the log_2 fold change in 5' P reads abundance for miscRNAs for RNase knockouts vs the wild type *B. subtilis* strain. **D**, as C but for 5' UTRs. **E**, Heat maps showing the strand-specific coverage of library size normalized 5' P reads centering around the end site (TES) of 5' UTRs (in this case the start codon) for *B. subtilis* control and different RNase knockouts. Each heat map row shows one 5' UTR. Dashed line indicates the start site of 5' UTRs (i.e., TSSs). **F**, as E, but for *E.coli*.

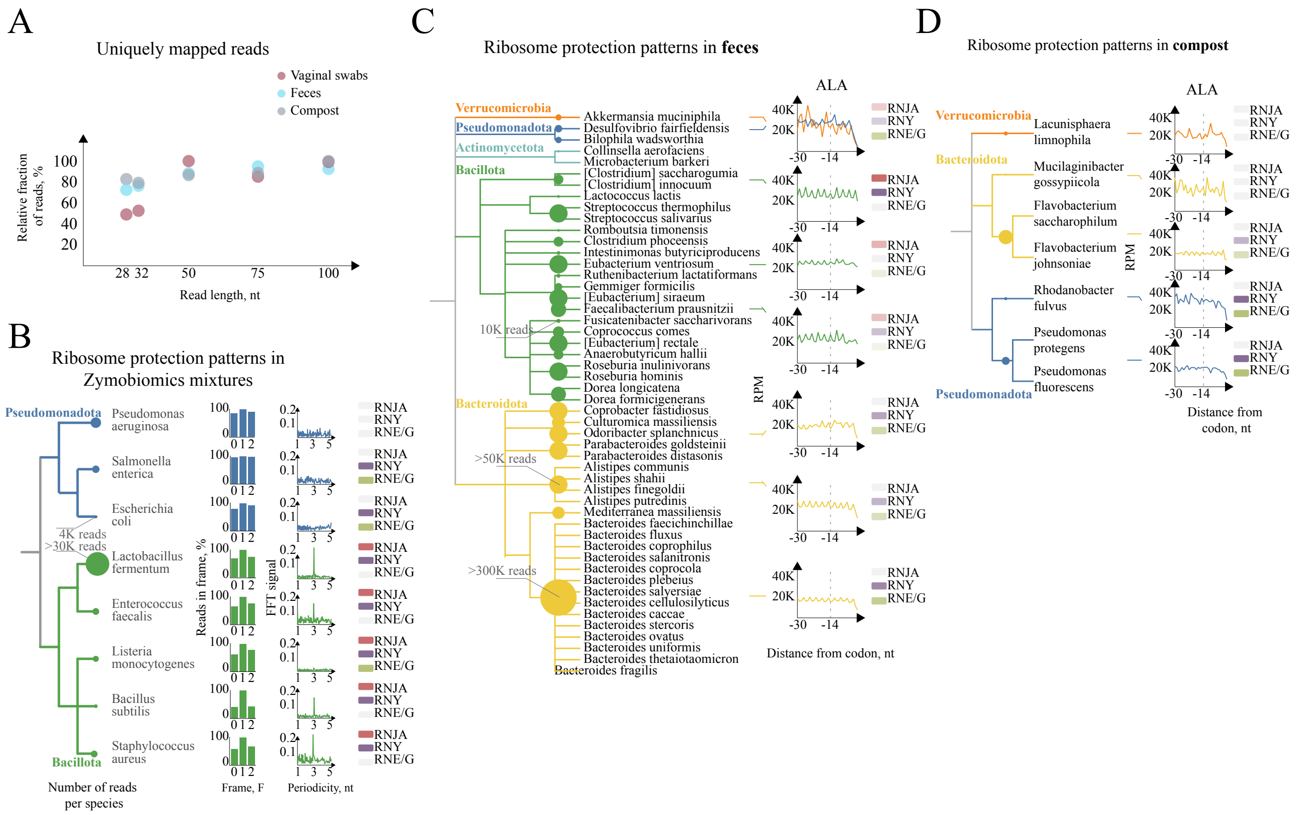

Extended Data Fig. 7. Longer ribosome protected 5PSeq reads enable better species-specific assignment in complex microbiomes. A, Relative number of uniquely mapped reads (scaled within each sample) as a function of used read length (trimmed computationally). **B**, 5PSeq analysis from frozen cell suspension from the ZymoBIOMICS Microbial Community Standard (intended for DNA analysis). The numbers of assigned reads to each species are marked in circles. Relative frame protection and Fast Fourier Transform (FFT), and presence/absence of enzymes as in Figure 1. **C,** 5PSeq analysis from fecal microbiomes. The numbers of assigned reads are marked in circles. Example of *in vivo* amino acid-specific (Ala) 3-nt ribosome protection periodicity for selected species. **D**, same for compost microbiome.

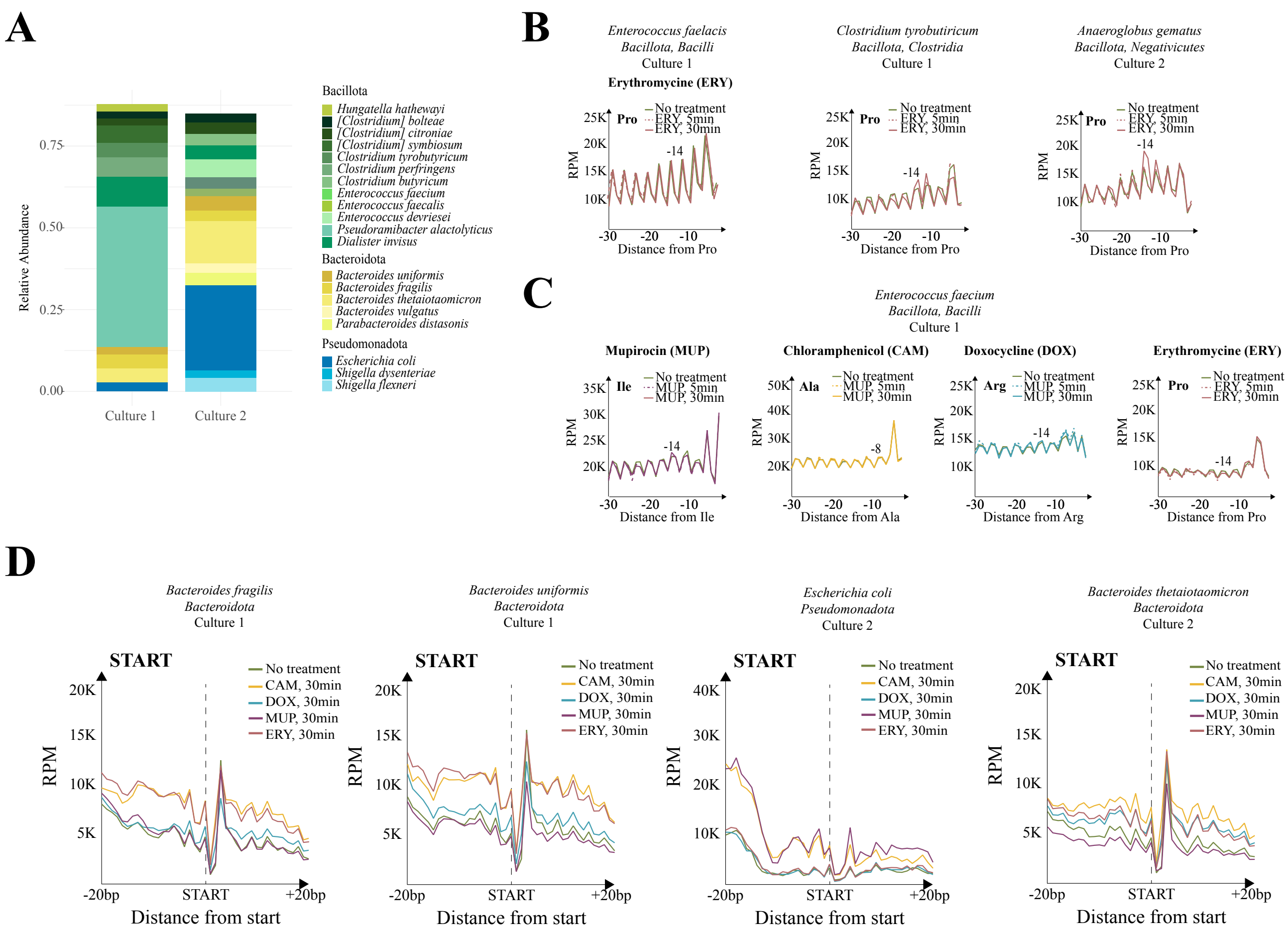

Extended Data Fig. 8. The composition of fecal cultures influences specie-specific response to antibiotic treatments. A, Relative composition of the used fecal cultures based on genomic DNA abundance. **B**, Line plots showing 5PSeq metagene abundance in respect to selected features illustrating species-specific differential response depending on the culture of origin. **C**, Example of the Bacillota *E. faecium* showing little response to the applied drug treatment. **D**, Bacteroidota species, lacking RNase J, also present clear alterations of the 5´P degradome profile in response to drug treatment.

Supplementary tables

Supplementary Table 1. Oligonucleotides used in this study.

Supplementary Table 2. Summary of samples and libraries analyzed in this study.

Supplementary Table 3. The $\log_2$ fold change values of the relative abundance of the reads undergoing degradation and frame protection index for each gene and Gene Ontology based gene set enrichment analysis in stress versus untreated conditions for *B. subtilis (subsp. subtilis 168 trpC2), E. coli (strain MG1655)* and *L. plantarum*. The enrichment p values are computed with a hypergeometric test and multiple test adjustment is performed with false discovery rate (FDR) with the R package WebGestaltR.

Supplementary Table 4. Effect of RNase knockouts on the abundance of RNA originating from different genomic features in B. subtilis. The table includes the $\log_2$ fold change for UTRs, TSS, coding region, miscRNAs and tRNAs.

Supplementary Table 5. The list of 5804 prokaryotic genome assemblies retrieved from NCBI and used for alignment.

Supplementary Table 6. List of species with relatively high coverage identified in all the studied samples.

Supplementary Table 7. Estimated expression of RNases using 5PSeq across the studied samples, in reads per million (RPM).

Uncropped scans of gels

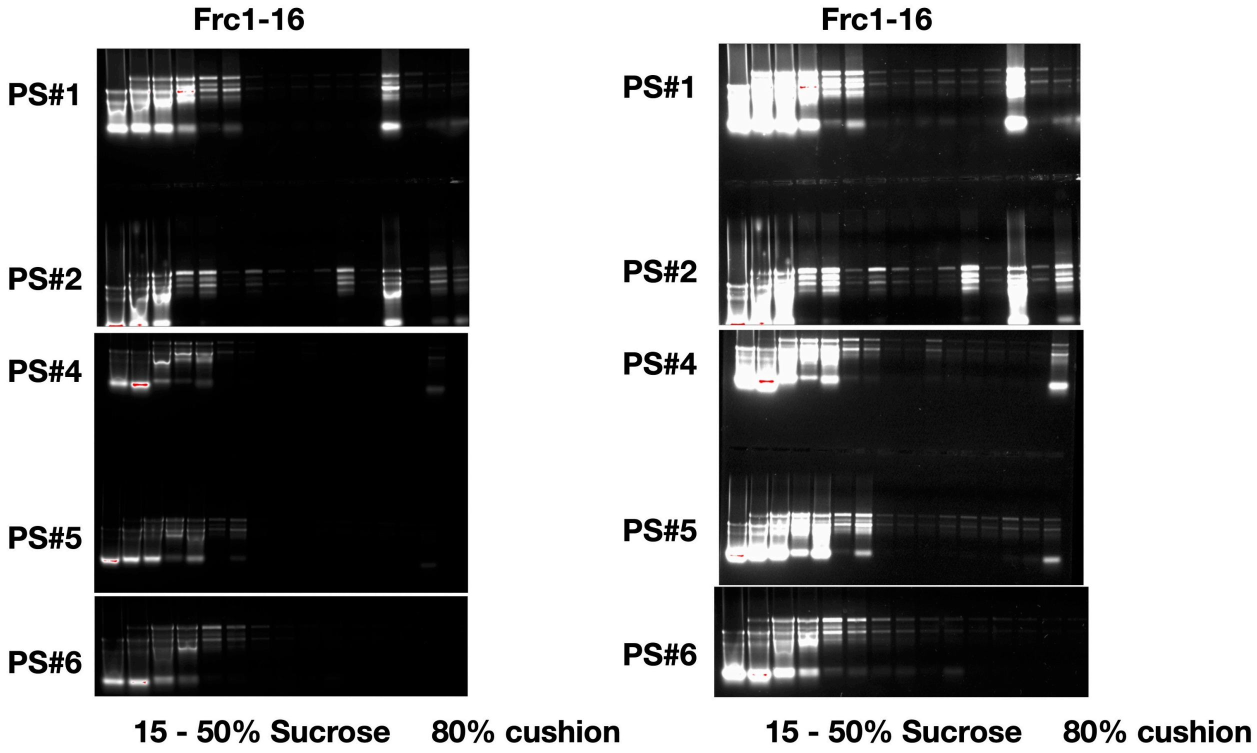

Polyribosome fractions of *B. subtilis* on 1.2% Aggarose gel TBE. PS#5 is depicted in Extended Data Fig. 1.
