# Supplementary material for: Atlas of mRNA translation and decay for bacteria
Source: Nat Microbiol. 2023 May 22;8(6):1123–36. doi: 10.1038/s41564-023-01393-z (PMC10234816; doi:10.1038/s41564-023-01393-z)
Supplement: Supplementary file 2 — Reporting Summary [file 41564_2023_1393_MOESM2_ESM.pdf]

nature portfolio

Corresponding author(s): Vicent Pelechano

Last updated by author(s): 22/03/23

Reporting Summary

Nature Portfolio wishes to improve the reproducibility of the work that we publish. This form provides structure for consistency and transparency in reporting. For further information on Nature Portfolio policies, see our Editorial Policies and the Editorial Policy Checklist.

Statistics

For all statistical analyses, confirm that the following items are present in the figure legend, table legend, main text, or Methods section.

n/a	Confirmed	
☐	☒	The exact sample size (*n*) for each experimental group/condition, given as a discrete number and unit of measurement
☐	☒	A statement on whether measurements were taken from distinct samples or whether the same sample was measured repeatedly
☐	☒	The statistical test(s) used AND whether they are one- or two-sided<br>*Only common tests should be described solely by name; describe more complex techniques in the Methods section.*
☐	☒	A description of all covariates tested
☐	☒	A description of any assumptions or corrections, such as tests of normality and adjustment for multiple comparisons
☒	☐	A full description of the statistical parameters including central tendency (e.g. means) or other basic estimates (e.g. regression coefficient) AND variation (e.g. standard deviation) or associated estimates of uncertainty (e.g. confidence intervals)
☐	☒	For null hypothesis testing, the test statistic (e.g. *F*, *t*, *r*) with confidence intervals, effect sizes, degrees of freedom and *P* value noted<br>*Give P values as exact values whenever suitable.*
☒	☐	For Bayesian analysis, information on the choice of priors and Markov chain Monte Carlo settings
☒	☐	For hierarchical and complex designs, identification of the appropriate level for tests and full reporting of outcomes
☒	☐	Estimates of effect sizes (e.g. Cohen's *d*, Pearson's *r*), indicating how they were calculated

Our web collection on statistics for biologists contains articles on many of the points above.

Software and code

Policy information about availability of computer code

Data collection	No software was used for data collection
Data analysis	All software used is described in the methods sections. The core of the analysis have been performed using our fivepeseq software v1.2 http://pelechanolab.com/software/fivepseq/ The code release for the fivepseq version 1.2.0 program is available at https://github.com/lilit-nersisyan/fivepseq/releases/tag/v1.2.0. The rest of the scripts and source data files to generate the figures are deposited in github at https://github.com/lilit-nersisyan/Atlas-of-mRNA-translation-and-decay-for-bacteria/, release version 1.0. (https://doi.org/10.17044/scilifelab.22305955)

For manuscripts utilizing custom algorithms or software that are central to the research but not yet described in published literature, software must be made available to editors and reviewers. We strongly encourage code deposition in a community repository (e.g. GitHub). See the Nature Portfolio guidelines for submitting code & software for further information.

Data

Policy information about availability of data

All manuscripts must include a data availability statement. This statement should provide the following information, where applicable:

- Accession codes, unique identifiers, or web links for publicly available datasets
- A description of any restrictions on data availability
- For clinical datasets or third party data, please ensure that the statement adheres to our policy

Sequencing data is deposited on GEO under accession number GSE153497. All the output files of fivepseq generated in this study are deposited at the SciLifeLab Data Repository https://doi.org/10.17044/scilifelab.22284709. Coverage tracks for vaginal samples are deposited at https://doi.org/10.17044/scilifelab.22305991

Human research participants

Policy information about studies involving human research participants and Sex and Gender in Research.

Reporting on sex and gender	Vaginal swabs were obtained from female donors. Faecal samples are deidentified and no information regarding sex or gender was recorded.
Population characteristics	Patient with cervical or head and neck cancer, with our with-out HPV infection. Samples were collected before and after anticancer treatment. For this study, we used anonymised information. Faecal samples are deidentified and no information was recorded.
Recruitment	Patient providing vaginal swabs after ethical consent were recruited in Stockholm, Uppsala, Norrland and Umeå. Faecal samples are deidentified and no information was recorded.
Ethics oversight	Collection and processing of sequenced vaginal samples was granted by the Regional Ethical Review Board in Stockholm (2017/725-31). Ethical approval for fecal samples and fecal cultures was waived by the review board as only deidentified samples from healthy donors were used and no samples were stored in a biobank.

Note that full information on the approval of the study protocol must also be provided in the manuscript.

Field-specific reporting

Please select the one below that is the best fit for your research. If you are not sure, read the appropriate sections before making your selection.

☒ Life sciences ☐ Behavioural & social sciences ☐ Ecological, evolutionary & environmental sciences

For a reference copy of the document with all sections, see nature.com/documents/nr-reporting-summary-flat.pdf

Life sciences study design

All studies must disclose on these points even when the disclosure is negative.

Sample size	We performed 2 to 5 biologically independent experiments (described in the methods section and GEO datasets). No sample size calculation was performed. In all cases investigated effect size measures was significantly bigger than the difference between biological replicates. Additionally, we asses reproducibility by comparing independent biological replicates.
Data exclusions	We only excluded samples not passing sequencing quality controls (e.g. to low number of reads mapping to the coding region). No other samples were excluded.
Replication	We performed multiple replicates at different times (over 3 years) and validated the results with multiple strains and species. All experiments providing sufficient number of reads were included and only experiments failing initial QC for sequencing quality were excluded. We used always biological independent replicates.
Randomization	We distribute samples and treatments across different experimental batches. This strategy was performed to avoid that batch effects could counfound any of the observed phenotypes
Blinding	No blinding was performed as is common in the field. Blinding does not provide additional certainly. Insted, we use different metrics to assure that samples traceability (e.g. checking genotype, clustering, PCA...).

Reporting for specific materials, systems and methods

We require information from authors about some types of materials, experimental systems and methods used in many studies. Here, indicate whether each material, system or method listed is relevant to your study. If you are not sure if a list item applies to your research, read the appropriate section before selecting a response.

Materials & experimental systems

n/a	Involved in the study
☒	☐ Antibodies
☒	☐ Eukaryotic cell lines
☒	☐ Palaeontology and archaeology
☒	☐ Animals and other organisms
☒	☐ Clinical data
☒	☐ Dual use research of concern

Methods

n/a	Involved in the study
☒	☐ ChIP-seq
☒	☐ Flow cytometry
☒	☐ MRI-based neuroimaging
